# Co-speciation and host-switching drives diversity of picornaviruses and sapoviruses in Malagasy fruit bats

**DOI:** 10.1038/s41598-025-34969-2

**Published:** 2026-01-23

**Authors:** Gwenddolen Kettenburg, Hafaliana C. Ranaivoson, Angelo Andrianiaina, Santino Andry, Amy R. Henry, Rachel L. Davis, Farida Laboune, Elizabeth R. Longtine, Sucheta Godbole, Sophia Horigan, Emily Cornelius Ruhs, Vololoniaina Raharinosy, Tsiry Hasina Randriambolamanantsoa, Vincent Lacoste, Jean-Michel Heraud, Philippe Dussart, Daniel C. Douek, Cara E. Brook

**Affiliations:** 1 Department of Ecology and Evolution, University of Chicago, Chicago, IL USA; 2 Department of Integrative Biology, University of California, Berkeley, CA USA; 3 Association Ekipa Fanihy, Antananarivo, Madagascar; 4 Department of Zoology and Animal Biodiversity, University of Antananarivo, Antananarivo, Madagascar; 5 Department of Entomology, University of Antananarivo, Antananarivo, Madagascar; 6 Human Immunology Section, Vaccine Research Center, NIAID, NIH, Bethesda, MD USA; 7 PREMISE, Vaccine Research Center, NIAID, NIH, Bethesda, MD USA; 8 Grainger Bioinformatics Center, Field Museum of Natural History, Chicago, IL USA; 9 Virology Unit, Institut Pasteur de Madagascar, Antananarivo, Madagascar; 10 Institut Pasteur à Paris, Paris, France; 11 Expertise France, Groupe Agence Française de Développement, Paris, France

**Keywords:** Ecology, Ecology, Evolution, Genetics, Microbiology, Zoology

## Abstract

Bats are reservoir hosts for numerous well-known zoonotic viruses, but their broader virus-hosting capacities remain understudied. *Picornavirales* are an order of enteric viruses that cause disease across a wide range of mammalian hosts, including Hepatitis A in humans and foot-and-mouth disease in ungulates. Host-switching and recombination drive the diversification of *Picornavirales* worldwide. *Picornaviridae* and *Caliciviridae* (families within *Picornavirales*) have been described in bats across mainland Africa, but surveillance for these viruses has been rare in the Southwest Indian Ocean Islands. Prior work in Madagascar has described numerous bat viruses, some with zoonotic potential, that demonstrate both high identity to and extreme divergence from viruses found in sister bat species in Africa. Using metagenomic Next Generation Sequencing of urine and fecal samples obtained from three species of endemic Malagasy fruit bats (*Eidolon dupreanum*, *Pteropus rufus*, and *Rousettus madagascariensis*), we identify and describe 13 full-length and 38 partial-length genomic sequences within the *Picornaviridae* and *Caliciviridae* families (36 picornavirus and 15 *Sapovirus* sequences). We find evidence that host-switching between Madagascar and mainland African bat picornaviruses and sapoviruses, followed by host-parasite co-speciation, likely shaped the diversification pattens of these novel sequences, with little evidence for cross-species transmission among Malagasy bat species in close contact.

**Supplementary Information:**

The online version contains supplementary material available at 10.1038/s41598-025-34969-2.

## Introduction

*Picornavirales* is a viral order associated with a large host range, spanning both plants and animals. Viruses in this order are characterized by a single-stranded, positive-sense RNA genome that forms non-enveloped icosahedral virions^1^ and can encode either one or two polyproteins^1^. Notably, viruses in two* Picornavirales* families, *Picornaviridae* and *Caliciviridae*, can infect some of the most common zoonotic hosts (bats, rodents, shrews)^2,3^, in addition to agriculturally significant hosts such as swine and cattle – both of which come into direct contact with humans^4–9^. Though some *Picornaviridae* and *Caliciviridae* viruses display close genetic similarity between those hosted by humans and animals, evidence of prior zoonosis is limited^9–13^. This diverse host range of *Picornaviridae* and *Caliciviridae* is partially due to recombination and host-switching events that drive virus evolution^14–19^.

Bats have garnered interest for their unique ability to host viruses known to be highly pathogenic in other mammals, including humans, without experiencing significant disease^20–25^. While bats are known to host viruses in the *Picornaviridae* and *Caliciviridae*^9,26–33^ families, these viruses are generally understudied compared with more well-known bat virus clades such as coronaviruses, filoviruses, lyssaviruses and paramyxoviruses. Previous work from Cameroon used metagenomic Next Generation Sequencing (mNGS) to describe diverse *Picornaviridae* and *Caliciviridae* in both *Eidolon helvum* and *Epomorphus gambianus* fruit bats, including novel kunsagiviruses and sapeloviruses that were divergent enough in amino acid composition to represent new species^30^. Divergent sapoviruses were also identified from the same bats^29^; in other cases, animal-derived sapoviruses have been shown to cluster closely with human-infecting genotypes^34–37^. In a separate study focusing on Algerian bats, evidence of past recombination events was detected within the genome of a novel mischivirus^28^. Host-virus coevolutionary analysis indicated that host-switching likely drove the diversification of this novel virus, in concordance with previously described patterns for the *Picornaviridae* family at large^28,38^. To date, the majority of bat virus work in these families has taken place in mainland Africa, with limited prior studies in the Southwest Indian Ocean Islands (SWIO: Madagascar, Seychelles, Mauritius, Réunion, and Comoros)^8^.

Of the SWIO, Madagascar boasts high levels of endemism and extraordinary evolutionary divergence among its flora and fauna due its isolation from mainland Africa and Asia for the past 80 million years^39^. Several evolutionarily distinct viruses have been previously identified in endemic Malagasy bats^40–46^ and lemurs^47^, matching expectations that the isolated evolutionary landscape leads not only to diverse hosts but also diverse viruses. However, research regarding bat-hosted *Picornaviridae* in Madagascar has been limited to only two prior studies, and no prior studies in *Caliciviridae*. One of these studies described a unique *Hepatovirus* in liver tissue collected from the Malagasy insectivorous bat, *Miniopterus cf. manavi*; sequence analysis of small mammal-hosted hepatoviruses broadly suggests some degree of host-virus co-evolution in their evolutionary history, with a hypothesized ancestral origin in bats and shrews^8^. In the other study, two full-length *Kobuvirus* sequences were detected via mNGS of fecal samples collected from *Eidolon dupreanum* fruit bats, forming a distinct clade basal to all other mammalian-hosted kobuviruses, further suggesting host-switching led to diversification of this genus^46^.

Here, we address a virus sampling gap in an evolutionarily distinct system (Madagascar) by contributing multiple novel genomes of traditionally undersampled bat-borne picornaviruses and sapoviruses. The viruses, recovered from three species of Malagasy fruit bat, *E. dupreanum*,* Pteropus rufus*, and *Rousettus madagascariensis*, display high identity to those in the same genera from sister bat species on the mainland African continent, with host-switching being the most likely evolutionary mechanism behind this diversification. However, these viruses appear to be species limited within Madagascar, with no virus sharing observed between all three fruit bat species, despite frequent co-roosting for *E. dupreanum* and *R. madagascariensis* and frequent contact at shared feeding sites for all three species.

## Materials and methods

### Sample collection

Longitudinal monthly sampling of three endemic Malagasy fruit bats (*E. dupreanum*,* P. rufus*, and *Rousettus madagascariensis*) was carried out from 2013 to 2019 at species-specific roost sites across Madagascar in part with an ongoing effort to investigate seasonal viral dynamics, as described previously^40,41,45,46,48^. Over this period, 2,156 bats were captured and processed under manual restraint^40,41,45,48^. Upon capture, bats were identified by species, age class, and sex, and individual fecal and urine swabs were collected. All excreta samples were collected in viral transport medium (VTM) and frozen in liquid nitrogen until samples could be processed. A subset of 810 (271 fecal/539 urine) samples collected between 2013 and 2019 at the following roost sites was used for the molecular analyses outlined here: district of Manjakandriana - Angavobe cave (-18.944 S, 47.949 E, *E. dupreanum)*; Angavokely cave (-18.933 S, 47.758 E, *E. dupreanum)*, district of Moramanga - Ambakoana roost (-18.513 S, 48.167 E, *P. rufus)*; Maromizaha cave (-18.9623 S, 48.4525 E, *R. madagascariensis)*, district of Ambilobe - Andrafiabe cave (-12.9435 S, 49.0555 E, *E. dupreanum* and *R. madagascariensis*); Cathedral cave (-12.952901 S, 49.046885 E, *E. dupreanum*); Antsiroandoha cave (-12.959336 S, 49.123698 E, *E. dupreanum*). This study was carried out in strict accordance with research permits obtained from the Madagascar Ministry of Forest and the Environment (permit numbers: 251/13, 166/14, 75/15, 92/16, 259/16, 019/18, 170/18, and 007/19) and under guidelines posted by the American Veterinary Medical Association. All field protocols employed were pre-approved by the UC Berkeley Animal Care and Use Committee (IACUC Protocol # AUP-2017-10-10393), and every effort was made to minimize discomfort to animals.

### Sample processing

Samples were frozen at the site of capture in liquid nitrogen and stored at -80 °C at the Virology Unit at the Institut Pasteur de Madagascar. Subsequently, RNA extraction of the samples was completed using the Zymo Quick DNA/RNA Microprep Plus kit (Zymo Research, Irvine, CA, USA), according to the manufacturer’s instructions and including the step for DNAse digestion. Extracted RNA was then shipped to either the Chan Zuckerberg Biohub (CZB; sample date range 2018–2019) (San Francisco, CA, USA) or the Vaccine Research Center (VRC), National Institute of Allergy and Infectious Diseases (NIAID), National Institutes of Health (NIH; sample date range 2013–2019) (Bethesda, USA) for mNGS.

Briefly, RNA underwent library preparation using the NEBNext Ultra II RNA Library Prep Kit (New England Biolabs, Beverly, MA, USA). The following modifications were made for CZB samples (date range 2018–2019): 25pg of External RNA Controls Consortium Spike-in mix (ERCCS, Thermo-Fisher) was added to each sample prior to RNA fragmentation; the input RNA mixture was fragmented for 8 min at 94 °C prior to reverse transcription; and a total of 14 cycles of PCR with dual-indexed TruSeq adapters was applied to amplify the resulting individual libraries. Quality was assessed by electrophoresis before performing large-scale paired-end sequencing (2 × 146 bp) on an Illumina NovaSeq (Illumina, San Diego, CA, USA). The following modifications were made for NIH samples (date range 2018–2019): input RNA was fragmented for 7 min at 94 °C prior to reverse transcription; and a total of 12 cycles of PCR were applied to amplify the resulting libraries. Quality was assessed by electrophoresis (Bioanalyzer 2100, Agilent) before paired-end sequencing (2 × 150 bp) on an Illumina NovaSeq 6000 (Illumina, San Diego, CA, USA).

### Virus detection

Raw reads were host-filtered, quality-filtered, and assembled on the Chan Zuckerberg Infectious Diseases (CZID) bioinformatics platform (v3.10 NR/NT 2019-12-01)^49^. A background profile named “bat” was created using all publicly available full-length bat genomes in GenBank at the time of sequencing (July 2019 for CZID samples, December 2023 for VRC samples) and used in host filtering. CZID results are outlined in Supplementary Table 1. CZID positive samples were verified using a command line BLAST pipeline, as described previously^40,41,45,46^. We used NCBI Virus taxids 12058/478825 for *Picornaviridae*/unclassified *Picornaviridae* and taxids 11974/179239 for *Caliciviridae*/unclassified *Caliciviridae* to develop nucleotide and protein libraries, against which we queried our novel sequences in command line BLASTn^50^ and BLASTx^50^ searches. With BLAST, we selected for contigs that showed significant nucleotide or protein BLAST alignment(s) (E-value < 0.00001 in addition to a bit score > 100 for protein BLAST) to *Picornaviridae* and *Caliciviridae* present in NCBI NR/NT database (v12-01-2019), then subset the hits to those with alignment length > 900/1000 nt/aa for nucleotide and protein BLAST, respectively. The following criteria were used to call “positive” samples following other virus discovery efforts from our group^40,41,45,46^: (1) CZID positive samples matched BLAST positive samples, and (2) contigs of interest had an average read depth of > 2 non-host reads per nucleotide.

### Genome annotation

Once contigs were identified as *Picornaviridae*/*Caliciviridae* sequences, we downloaded annotated reference *Picornaviridae*/*Caliciviridae* sequences from NCBI and aligned to our novel sequences using MAFFT^51^ (v7.450) with default settings in Geneious Prime (v08-18-2022). We annotated the polyprotein and peptide regions using available *Picornaviridae*/*Caliciviridae* reference sequences that corresponded to the top BLAST hit. Reference sequences were also used to identify potential cleavage sites and conserved motifs within RNA-dependent RNA polymerase (RdRp), polyprotein major proteases, and NTPase-helicase. Contigs were then sorted into the following virus genera: *Picornaviridae* – unclassified bat picornavirus, *Cardiovirus*, *Hepatovirus*, *Kobuvirus*, *Kunsagivirus*, *Mischivirus*, *Sapelovirus*, and *Teschovirus*; *Caliciviridae* – *Sapovirus*.

### Phylogenetic analysis

We constructed 10 maximum-likelihood (ML) nucleotide phylogenetic trees using IQ-TREE^52^, representing all genera in which we identified at least one novel sequence > 2000 bp in length. 1000 ultrafast bootstraps for each tree were run in UFBoot2^53^, and we allowed IQ-TREE to test and select the best nucleotide model using ModelFinder^54^. All trees were rooted using an outgroup of Sindbis virus (accession NC_001547). We first constructed (1) a phylogeny encompassing the conserved polymerase (3D peptide) region and included global background sequences from viral genera corresponding to those clades represented by all novel Madagascar sequences (unclassified bat picornavirus, *Cardiovirus*, *Hepatovirus*, *Kobuvirus*, *Kunsagivirus*, *Mischivirus*, *Sapelovirus*, *Teschovirus*, and *Sapovirus*). Then, we constructed nine additional ML trees using full-length sequences from the following viral genera as references: (2) *Cardiovirus*, (3) *Hepatovirus*, (4) *Kobuvirus*, (5) *Kunsagivirus*, (6) *Mischivirus*, (7) *Shanbavirus*/unclassified bat picornavirus, (8) *Sapelovirus*, (9) *Teschovirus*, and (10) *Sapovirus*. Reference sequences and novel sequences were aligned using MAFFT^51^. Visualization of phylogenetic trees was performed using the ggtree^55^ package in R. We further detail NCBI virus taxid information, best fit substitution models, alignment names, and size of overlap region per phylogeny in Supplementary Table 2.

### Similarity analysis

We conducted similarity analyses, using PySimPlot^56^, on all nucleotide and translated amino acid full-length sequences. PySimPlot^56^ commands were formulated using our novel Madagascar sequences as the query sequences; in cases where multiple novel sequences were identified within a single genus, we input one of those sequences as the query and included the others within the reference sequences. Otherwise, reference sequences for input to PySimPlot^56^ were comprised of the top three full-genome BLASTx hits for the query. In all cases, sequences were aligned in MAFFT^51^, and both similarity analyses were carried out using a window size of 100aa and a step size of 20aa for amino acid comparisons and a window size of 100 bp and a step size of 1 bp for nucleotide comparisons. Genomic areas of interest for host interactions and immunogenicity as defined by the literature were highlighted in the plots as well (2A and 3A peptides^19,57^ for *Picornaviridae*, NS4 and VP1 peptides^58,59^ for sapoviruses).

### Co-evolutionary analysis

We used Jane v4.0^60^, which examines congruence of the least costly topological reconstruction of the relationship between host and parasite trees, to interrogate the extent to which co-speciation vs. host-switching appeared to underly the relationship between novel viruses and their hosts. Jane examines four evolutionary events: co-speciation (vertex on host tree is associated with vertex on parasite tree), duplication (parasite lineage speciated independently of the host lineage and remained with the host), duplication and host-switching (parasite lineage speciated but one parasite switched to another host), and loss (the path between a parasite vertex and its child passed through a host vertex)^60,61^.

Using previously described Jane parameters for event costs in bat picornaviruses^28^, we tested three scenarios: a null expectation model of all events set to 0, a complex speciation model of all events set to 1, and a co-speciation model in which all events were set to 1 except for co-speciation which was set to 0, thus biasing the model to favor cophylogenetic pathways. Population size and generation parameters were left at program defaults of 100 and 100, values previously used in multiple bat-parasite coevolutionary analyses^28,62,63^. Initially, we also explored simulations including smaller population sizes paired with relatively larger generation numbers as originally recommended by the developers of Jane^60^. However, these changes did little to alter our downstream inference, and as a result, we report findings here from the default parameter values only. If the least costly solution resolved as the second scenario (with all events set to 1), then we assumed host-switching and duplications to be the most likely evolutionary scenario, and if the least costly solution was the third scenario (with all events set to 1 except for co-speciation), we assumed co-speciation to be the most likely evolutionary scenario. Host phylogenies were acquired from published sources^64^. Co-evolutionary relationships were plotted as tanglegrams using ‘cophylo’ in the R package ‘phytools’^65^.

### RDP4 recombination analysis

Recombination Detection Program 4 (RDP4)^66^ was used to evaluate signals of recombination between our novel sequences and those most phylogenetically related to the novel sequences, and between the novel sequences themselves. Six default recombination analyses within RDP4 (RDP, GENECONV, Bootscan, MaxChi, Chimaera, and 3Seq) were used to estimate likely recombination events and the location of the recombination event within all full-length sequences identified in this study. Using default settings for all six algorithms, we considered recombination events to be reliable if at least five out of six analyses were significant with a cutoff *P*-value of 0.05. As described in the similarity analysis methods section, we highlighted genomic regions of interest for host interactions and immunogenicity in these plots as well.

## Results

### Sampling, genome classification and roost-site diversity

#### Sampling

We sequenced RNA extracted from 810 (271 fecal and 539 urine) samples from 803 individual bats (373 *E. dupreanum*, 146 *P. rufus*, and 284 *R. madagascariensis*). In total, 23/803 bats were *Picornaviridae* positive (2.86%), and 9/803 bats were *Caliciviridae* positive (1.12%) (Supplementary Fig. 1, Table 1). Of the 23 *Picornaviridae* positive bats, 13 were *E. dupreanum*, 1 was *P. rufus*, and 9 were *R. madagascariensis*. The 9 *Caliciviridae* positive bats were near-evenly split between *E. dupreanum* (4) and *R. madagascariensis* (5) with no positives identified in *P. rufus*. No bats were positive at the Ankarana caves (*N* = 151). *Picornaviridae* and *Caliciviridae* prevalence was highest in *E. dupreanum* at the Angavokely/Angavobe roosts (13/281 bats *Picornaviridae* and 4/281 bats *Caliciviridae*) with a similar prevalence reported in *R. madagascariensis* at the Maromizaha roost (9/225 bats *Picornaviridae* and 5/225 bats *Caliciviridae*) (Supplementary Fig. 1, Table 1). In *P. rufus*, only one bat out of 146 individuals was found to be positive for *Picornaviridae* (Supplementary Fig.1, Table 1).

**Table 1:** Sampling efforts by roost site and species, in addition to breakdown by *Picornaviridae* and *Caliciviridae* positives. An asterisk denotes that one *R. madagascariensis* was positive for both *Picornaviridae* and *Caliciviridae*.

District	Roost site	Species	# Tested bats	*Picornaviridae* positive bats	*Caliciviridae* positive bats
Ambilobe, Madagascar	Ankarana caves (Andrafiabe, Cathedral, Antisiroandoha)	*E. dupreanum*	92	0	0
		*R. madagascariensis*	59	0	0
Moramanga, Madagascar	Ambakoana roost	*P. rufus*	146	1 (0.68%)	0
	Maromizaha cave	*R. madagascariensis*	225	9* (4.00%)	5* (2.22%)
Manjakandriana, Madagascar	Angavobe/Angavokely caves	*E. dupreanum*	281	16 (5.69%)	4 (1.42%)

#### Genome classification

Of the positive bats, we found an average of ~ 50,000,000 total reads across all samples, with a range of ~ 76–95% passing quality control (QC) (Supplementary Table 1). Despite slightly different processing methods between samples sequenced at CZB vs. NIH (ERCCS was used in CZB processing only), we found no discernable difference in QC between samples processed with ERCCS and without, with ~ 89% and ~ 90% respectively passing QC (Supplementary Table 1). In general, there were more reads mapping to *Picornaviridae* than *Caliciviridae* as well, reflecting our higher number of *Picornaviridae* contigs versus *Caliciviridae* contigs (Supplementary Table 1). From positive bats, we recovered 13 full-length (12 *Picornaviridae* and 1 *Caliciviridae*) and 38 partial-length (24 *Picornaviridae* and 14 *Caliciviridae*) sequences primarily from *E. dupreanum* (23 *Picornaviridae* from 13 individuals and 5 *Caliciviridae* from 4 individuals) and *R. madagascariensis* (12 *Picornaviridae* from 9 individuals and 10 *Caliciviridae* from 5 individuals) (Supplementary Tables 3 and 4). Only one viral *Picornaviridae* sequence (full-genome) was recovered from an individual *P. rufus* (Supplementary Tables 3 and 4). Using BLAST^50^, sequences within *Picornaviridae* corresponded to the following viral genera: *Cardiovirus* (1 sequence from 1 viral species), *Hepatovirus* (4 sequences from 1 viral species), *Kobuvirus* (6 sequences from 1 viral species), *Kunsagivirus* (1 sequence from 1 viral species), *Mischivirus* (1 sequence from 1 viral species), *Sapelovirus* (11 sequences from 3 viral species), *Teschovirus* (4 sequences from 3 viral species), and unclassified “bat picornavirus” (8 sequences from 4 viral species) (Supplementary Table 3). *Caliciviridae* was only represented by a single genus: *Sapovirus* (15 sequences from 8 viral species) (Supplementary Table 4). In full genomes, average support was > 88 reads/base pair, and in partial genomes, average support was > 1764 reads/base (Supplementary Figs. 2 and 3).

Some *Picornaviridae* genera were only found in *E. dupreanum* (*Cardiovirus*, *Hepatovirus*, *Kobuvirus*, and *Kunsagivirus*) (Supplementary Table 3), with highest identity to *Picornaviridae* identified from its sister species *E. helvum* in Ghana and Cameroon (Supplementary Table 3), a host widely distributed across the African continent but absent from Madagascar^67^. Exceptions to this pattern included an *E. dupreanum*-hosted *Cardiovirus* sequence which had the highest identity to a divergent encephalomyocarditis virus isolated from an orangutan from Singapore^68^, several *Kobuvirus* sequences which had highest identity to a *E. dupreanum*-hosted *Kobuvirus* from a previous study^46^, and a *Teschovirus* sequence with highest identity to a Ugandan Rousettus aegypticus bat teschovirus^69^. *Cardiovirus* has been detected in bats before, namely East Asian *Miniopterus fuliginosus* bats^70^, but the lack of publicly-available sequences from this previous study precludes sequence comparison with our novel Malagasy bat *Cardiovirus*.

One *Picornaviridae* genus, *Mischivirus*, was found only in a single *P. rufus* (Supplementary Table 3). This novel sequence showed very low identity (46% over 81% genome coverage) to its closest match (mischivirus C1 from a *Hipposideros gigas* bat from the Democratic Republic of the Congo) (Supplementary Table 3).

Among *Picornaviridae* genera exclusively hosted by *R. madagascariensis* (unclassified bat picornavirus), and those hosted by both *E. dupreanum* and *R. madagascariensis* (*Sapelovirus* and *Teschovirus*), we identified numerous viruses with high identity to viruses hosted by sister species *R. aegypticus*^71^ sampled in Uganda and Kenya. The unclassified bat picornaviruses were most closely related to other previously-described “bat picornaviruses”^7^ but, within this clade, form what appears to be a largely divergent *Picornaviridae* genus with an average BLASTx identity of ~ 80% with ~ 90% genome coverage to the closest match (Supplementary Table 3). *R. madagascariensis*-hosted *Sapelovirus* and *Teschovirus* had highest identity to East African (Uganda and Kenya) *R. aegypticus*-hosted *Sapelovirus* and *Teschovirus* sequences^69^ (Supplementary Table 3).

The representative *Caliciviridae* genus, *Sapovirus*, was found in both *E. dupreanum* and *R. madagascariensis* (Supplementary Table 4). *Sapovirus* sequences from *E. dupreanum* and *R. madagascariensis* showed highest identity to a Cameroonian *E. helvum*-hosted *Sapovirus*^29^ and East African *R. aegypticus*-hosted sapoviruses^69^, respectively (Supplementary Table 4).

#### Geographic and host characteristics of sequences

**Fig. 1:**
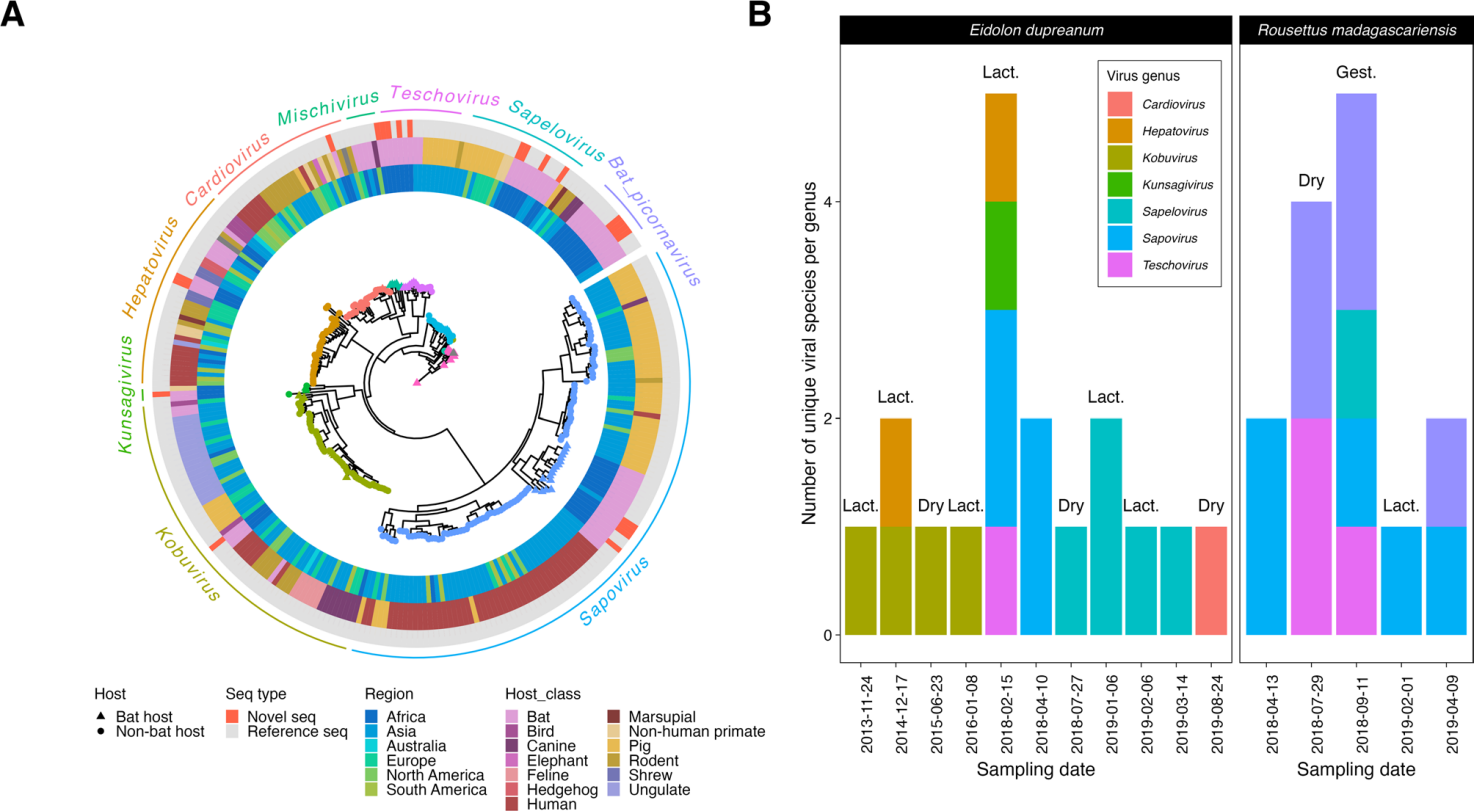
(**A**) Maximum-likelihood nucleotide phylogeny (IQ-TREE^52^) of sequences from *Picornaviridae* and *Caliciviridae* genera from which novel sequences were classified from the RdRp region of the genome, using a best-fit TIM2 + F + R10 nucleotide substitution model (Supplementary Table 2). 1000 ultrafast bootstraps were computed using UFBoot2^53^. Rings indicate region (blue scale, inner ring) and host class (warm scale, middle ring) for each sequence. Novel sequences are indicated by a red square (outer ring). Tip shape indicates host in which the sequence was derived corresponding to the legend (bat vs. non-bat). Tree is rooted in Sindbis virus (accession NC_001547.1), which has been removed for ease of visualization. Branch lengths are scaled by nucleotide substitutions per site. (**B**) Summary bar plot of diversity of unique species per viral genera (colors) identified from *E. dupreanum* (Angavokely/Angavobe caves) and *R. madagascariensis* (Maromizaha cave) bats caught within the same sampling date. *P. rufus* (Ambakoana roost) was excluded due to only having one identified novel virus in a single bat. Seasonal events such as gestation, lactation, and the dry season are abbreviated and identified above each bar, unless the sampling date does not fall within a defined event.

After identification of viral genera and species through BLAST (Supplementary Tables 3 and 4), we constructed an RdRp phylogeny which showed phylogenetic clustering of the novel Malagasy bat picornaviruses and sapoviruses with other reference sequences, allowing further virus classification (Fig. 1A). Novel sequences all generally clustered with African sequences and other bat-hosted sequences (Fig. 1A).

#### Roost site diversity

We identified up to 5 different viral species sourced from the same group of bats captured within a single sampling session (Fig. 1B). Overall, the *E. dupreanum* Angavokely roost was represented the most frequently in the samples analyzed for this study and demonstrated the most unique viruses overall (Fig. 1B and Supplementary Tables 3 and 4), but the *R. madagascariensis* Maromizaha roost also demonstrated high virus diversity within fewer viral genera – with multiple repeats of the same virus genotype recovered from different individuals (Fig. 1B). The highest diversity was usually observed during the reproductively stressful seasons of lactation for *E. dupreanum* and gestation for *R. madagascariensis* (Fig. 1B).

### Genome annotation

#### Picornaviruses

For all new picornaviruses, we successfully annotated a single ORF-encoding polyprotein which includes the P1 region of structural polypeptides and the P2/P3 regions of replication-associated nonstructural polypeptides. We further identified cleavage sites within the polyprotein between each peptide of the P1 region (L, VP4, VP2, VP3, VP1), the P2 region (2A, 2B, 2C) and the P3 region (3A, 3B, 3C, and the RNA-dependent-RNA-polymerase [RdRp] 3D) (Supplementary Table 5). Following previous work^27,30,72^, we identified the conserved motifs, helicase GxxGxGKS, 2A protease GxCG, 3C protease GxCG, and RdRp motifs KDELR, YGDD, and FLKR (Supplementary Table 6).

#### Sapoviruses

In all *Sapovirus* sequences, we identified and annotated the ORF1-encoding polyprotein in addition to ORF2. Within the ORF1 polyprotein, we further identified cleavage sites between peptides NS1/NS2, Helicase, NS4, Vpg, Pro-Pol (RdRp), and VP1 in addition to annotating the small structural protein VP2 encoded by ORF2 (Supplementary Table 7). Again following prior work^29^, we identified conserved helicase GxPGxGKx, Vpg KGKTK and DDEYDE, protease GxCG, RdRp conserved WKGL, KDELR, DYSKWDST, GLPSG, and YGDD, and finally VP1, PPG, and GWS motifs (Supplementary Table 8).

### Genus-specific phylogenetic analysis

#### Picornaviruses

**Fig. 2:**
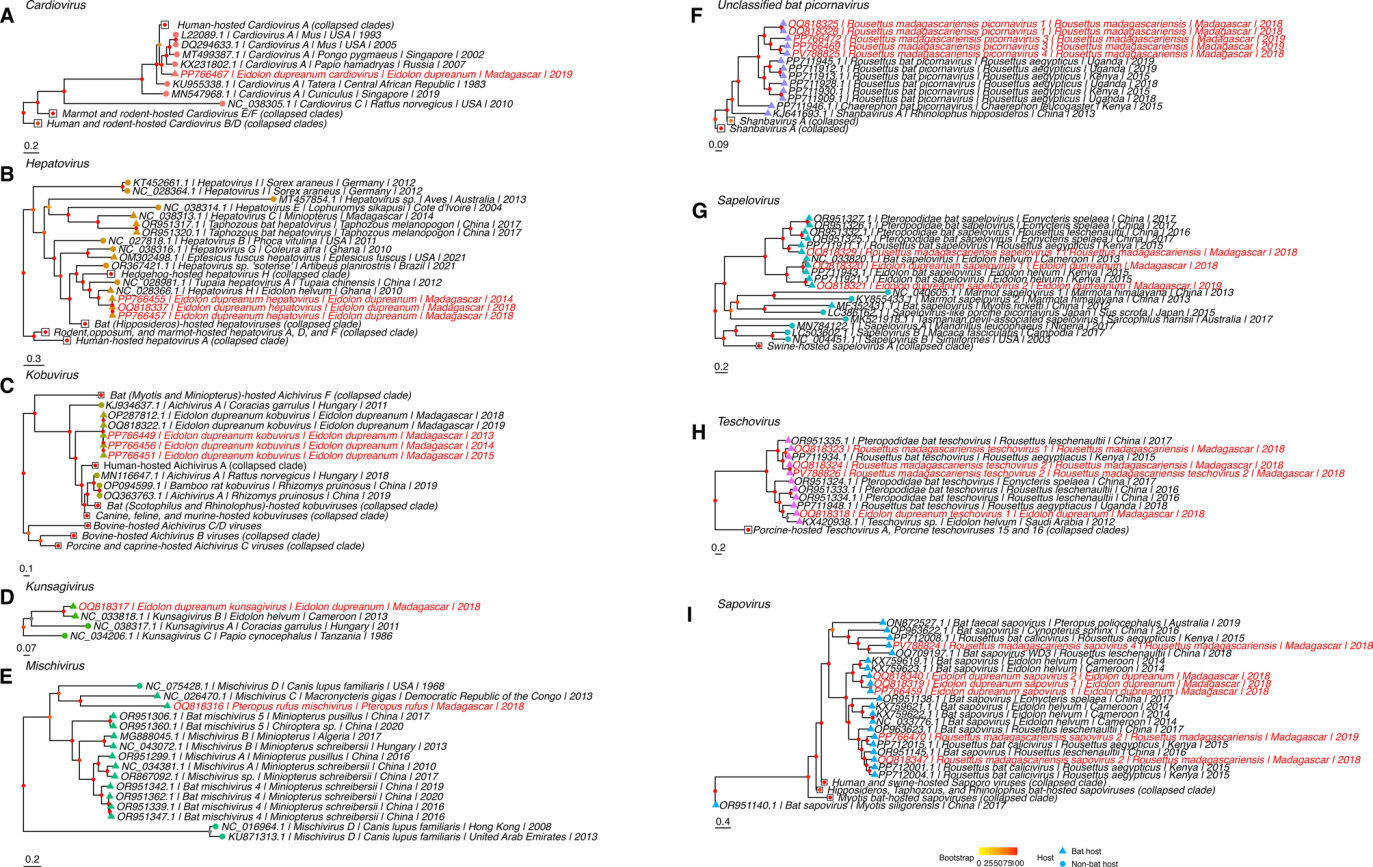
Maximum-likelihood nucleotide phylogenies across the whole genome (IQ-TREE^52)^ of (**A**) *Cardiovirus* sequences, (**B**) *Hepatovirus* sequences, (**C**) *Kobuvirus* sequences, (**D**) *Kunsagivirus* sequences, (**E**) *Mischivirus* sequences, (**F**) *Shanbavirus*/unclassified bat picornavirus sequence, (**G**) *Sapelovirus* sequences, (**H**) *Teschovirus* sequences, and (**I**) *Sapovirus* sequences. Best fit nucleotide substitution models and overlapping base pair length per tree are summarized in Supplementary Table 2. 1000 ultrafast bootstraps were computed using UFBoot2^53^ and are visualized on tree branches. Novel sequences are highlighted in yellow. Collapsed clades are represented by white squares. Tip shape indicates host in which the sequence was derived corresponding to the legend. Trees are rooted in Sindbis virus (accession NC_001547.1). Roots have been removed for ease of visualization. Branch lengths are scaled by nucleotide substitutions per site, corresponding to each scalebar.

The partial E. dupreanum cardiovirus sequence had high identity to a divergent orangutan-hosted encephalomyocarditis virus (Supplementary Table 3) and appeared to be phylogenetically basal to these primate-hosted cardioviruses. Due to the novelty of cardioviruses in bats, addition of more sequences would likely resolve the placement of this sequence in its own clade (Fig. 2A). E. dupreanum hepatovirus sequences formed a clade sister to a Cameroonian *E. helvum*-hosted *Hepatovirus* (Fig. 2B). As mentioned previously, the E. dupreanum kobuvirus sequences described in this paper were determined to be genetic variants of the same virus previously described^46^, although the addition of more novel sequences provides support for the previous observation that *E. dupreanum*-hosted kobuviruses form a basal clade to other human, bat, and mammal-hosted kobuviruses, suggesting a possible role for host-switching (Fig. 2C). Few full-length *Kunsagivirus* sequences are published, but phylogenetically, E. dupreanum kunsagivirus formed a monophyletic clade with a Cameroonian *E. helvum*-hosted *kunsagivirus* B, separate from the *Kunsagivirus* sequences found in other mammalian hosts (Fig. 2D).

The most divergent virus, P. rufus mischivirus, formed a monophyletic clade with a *H. gigas mischivirus C* sequence that is separate from all other bat-hosted mischiviruses that have been previously identified in *Miniopterus* spp. (Fig. 2E). The long branch length separating these clades suggests considerable differences in nucleotide substitution rates as well (Fig. 2E). Without more sequences to resolve the phylogeny, it is unclear that geographical species distribution alone may be driving viral species differentiation. *H. gigas* is part of the family *Hipposideridae*, within the *Yinpterochiroptera* suborder, which also includes *P. rufus*. By contrast, *Miniopterus* spp. are part of the *Yangochiroptera* suborder^74^. While these patterns suggest a co-speciation of viruses along host evolutionary lines, previous analyses have instead supported host-jumping mechanisms as a driver of *Mischivirus* diversity in bats^28^.

*R. madagascariensis*-hosted picornaviruses formed a sister paraphyletic clade to other *Rousettus* bat picornaviruses from East Africa, and within their own clade, separated into two smaller clades – R. madagascariensis picornavirus 1 in one clade and R. madagascariensis picornaviruses 3 and 4 in another (Fig. 2F). R. madagascariensis picornavirus 2 was a short sequence (< 2000 bp in length) and was therefore excluded from phylogenetic analysis. Most bat sapeloviruses form a monophyletic clade sister to marmot and Tasmanian-devil sapeloviruses (excepting one *Myotis*-hosted sapelovirus which falls within the non-bat clade); simian and swine-hosted sapeloviruses resolved basal in the phylogeny (Fig. 2G). Within the larger bat sapelovirus clade, two sister clades formed between *Eidolon*-hosted sapeloviruses and *Rousettus*/*Eonycteris*-hosted sapeloviruses (Fig. 2G). As seen with BLAST, E. dupreanum sapeloviruses 1 and 2, and R. madagascariensis sapelovirus 1 had respectively high identity to those viruses in Africa hosted by sister bat species (Fig. 2G, Supplementary Table 3). In a similar pattern as the *Sapelovirus* phylogeny, bat teschoviruses formed a divergent and distinct clade to those hosted by swine (porcine) – which are the usual hosts of teschoviruses^75^ (Fig. 2H). Within the bat-hosted teschoviruses, viruses again clustered following species phylogenetics: R. madagascariensis teschovirus 1, R. madagascariensis teschovirus 2, and E. dupreanum teschovirus 1 grouped with similar viruses hosted by other bat species within the same respective genera (Fig. 2H).

Generally, in virus clades with prior, publicly-reported viruses for comparison, Malagasy bat picornaviruses from *E. dupreanum* and *R. madagascariensis* were, respectively, phylogenetically closest to *E. helvum* and *R. aegypticus*-hosted picornaviruses described from mainland Africa (Fig. 2).

#### Sapoviruses

Bat sapoviruses are phylogenetically distinct from human and swine-hosted Sapporo viruses, which also fall within the *Sapovirus* genus (Fig. 2I). In our analyses, most bat sapoviruses formed a monophyletic clade proximal to the human and swine Sapporo viruses, though *Myotis* spp. - hosted sapoviruses resolved as basal in the entire *Sapovirus* clade. Novel *E. dupreanum* and *R. madagascariensis*-hosted sapoviruses again nested sister to closely related African *E. helvum* and *R. aegypticus*-hosted sapoviruses, respectively (Fig. 2I), following similar host-specific virus diversification seen in the picornaviruses.

### Similarity analysis across the genome

#### Picornaviruses

**Fig. 3:**
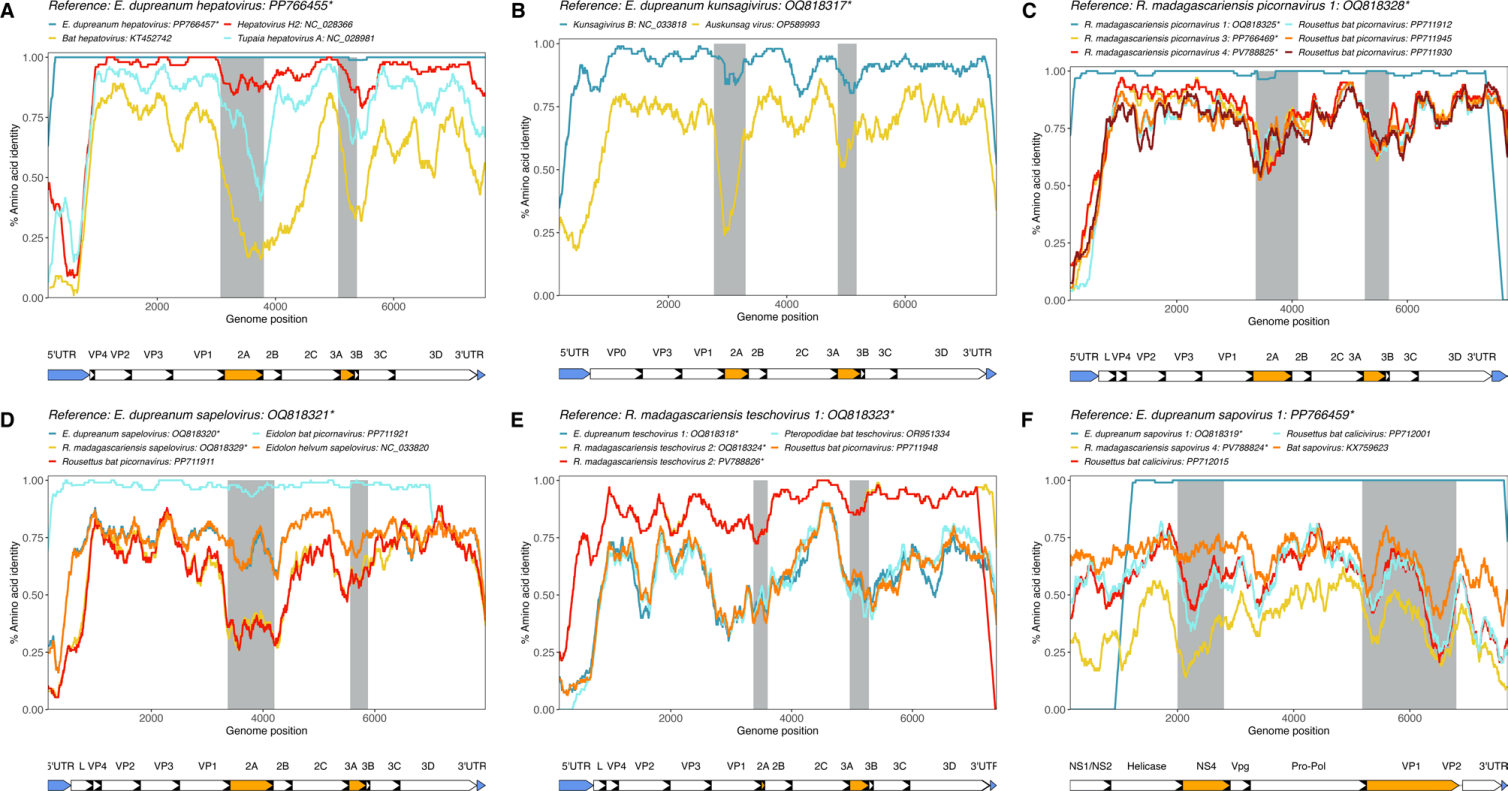
Amino acid similarity computed in PySimPlot^56^ for novel full-length sequences. Similarity analyses with query sequence (**A**) E. dupreanum hepatovirus: accession PP766455, (**B**) E. dupreanum kunsagivirus: accession OQ818217, (**C**) R. madagascariensis picornavirus 1: accession OQ818328, (**D**) E. dupreanum sapelovirus: accession OQ818321, (**E**) R. madagascariensis teschovirus 1: accession OQ818323, and (**F**) E. dupreanum sapovirus 1: accession PP766459 against similar sequences identified from BLAST and other matched novel sequences within the same genus. Novel sequences described in this study are starred with an asterisk. Line color corresponds to different virus sequences, with annotated regions of the genome below each plot. Plots were generated with a window size of 100aa and a step size of 20aa. Peptides in orange and corresponding grey shaded areas denote areas of interest for host interactions and immunogenicity, and blue peptides denote 5’ and 3’ UTRs. Amino acid similarity plots for *Kobuvirus* and *Mischivirus* are in Supplementary Fig. 4. Matched nucleotide similarity plots are in Supplementary Fig. 5.

Many novel sequences had the highest identity (average > 80% BLASTx^50^ identity) to African *E. helvum* and *R. aegypticus* – hosted viruses (Supplementary Table 3), which are sister host species to *E. dupreanum* and *R. madagascariensis* respectively. When comparing Madagascar sequences against their closest matches in GenBank, we observed a consistent sharp drop in similarity in the 2A-to-2B and 3A-to-3B broad peptide regions of the genome (Fig. 3A-E, Supplementary Fig. 4 for amino acid similarity plots, Supplementary Fig. 5 for nucleotide similarity plots). The border of the P1 and P2 regions (between VP1 and 2A, respectively) are thought to comprise a genomic region susceptible to recombination^19^, while the 3A region of *Picornaviridae*, known to be highly divergent across genera, is associated with host range determination and viral replication^57^, thus offering some explanation for the heightened genomic divergence in these regions. We also consistently observed drops in similarity in the 5’ and 3’-UTRs (Fig. 3A-E, Supplementary Figs. 4 and 5). The 5’-UTR of *Picornaviridae* is thought to play a role in antagonizing innate host immunity; indeed, work in enteroviruses shows that the development of mutations in this region can dampen replication competence of the virus^76^. As the 5’ UTRs of most of the novel Malagasy *Picornavirales* demonstrated very low similarity (< 50%) to related reference sequences (Fig. 3A-E), it is possible that these Malagasy bat viruses employ different replication and immune evasion strategies than previously documented (Fig. 3A-E).

E. dupreanum hepatovirus sequences were nearly identical to each other, and across the genome, demonstrated highest identity to *Hepatovirus* H2, from a *E. helvum* bat (Fig. 3A). Nonetheless, the Malagasy viruses showed a large dip in identity to the *E. helvum* virus in the 5’-UTR (< 25% average identity), in addition to other dips across the 2A/2B peptides, the 3A/3B peptides, and within the 3’ UTR (Fig. 3A). E. dupreanum kunsagivirus mirrored these patterns, showing reduced identity to previously described kunsagiviruses in the 5’-UTR, 2A, 3A, and 3’-UTR regions, in addition to a slight reduction in identity in the 2C region (Fig. 3B). E. dupreanum kobuvirus sequences described in this study were nearly identical across the genome to an E. dupreanum kobuvirus sequence described previously^46^ (Supplementary Fig. 4A). P. rufus mischivirus had low identity to comparable sequences across the genome (< 75%), with more dramatic drops observed in the 5’-UTR and the 3A peptide regions (Supplementary Fig. 4B).

R. madagascariensis picornavirus 1 sequences had high (~ 99%) identity across the genome to each other, and R. madagascariensis picornavirus 3 and 4 were most like African *Rousettus*-hosted bat picornaviruses (Fig. 3C). Though R. madagascariensis picornaviruses 1, 3, and 4 were derived from the same host species, they nonetheless demonstrated < 80% average identity to one another and demonstrated predictable divergence in the 2A, 3A, and 5’ and 3’-UTR regions, suggesting that these viruses likely have different replication and immune evasion strategies (Fig. 3C). Like R. madagascariensis picornaviruses, the novel *E. dupreanum* and *R. madagascariensis-*hosted *sapeloviruses* had high identity to sister species (e.g. *E. helvum* and *R. aegypticus*, respectively)-hosted viruses, but still differed in 5’-UTR, 2A peptide, and 3A peptide regions (Fig. 3D). R. madagascariensis teschovirus 1 was not as closely related to R. madagascariensis teschovirus 2 (Fig. 3E). However, E. dupreanum teschovirus 1 had highest identity to Ugandan *R. aegypticus*-hosted teschoviruses (Fig. 3E). As before, we observed drops in similarity between the Malagasy bat teschoviruses and previously described sequences in the 3A peptide region, in addition to drops in similarity in VP2 and VP1 regions, as well (Fig. 3E).

#### Sapoviruses

For sapoviruses, we anticipated lower similarity in the VP1, NS4, and Vpg regions of the genome. VP1 is associated with host interactions and immunogenicity^58^, while *Caliciviridae* NS4 has been suggested to be a homolog for *Picornaviridae* 3A, since both families exhibit similar genome organization^59^. If true, then divergence in NS4 might be expected due to divergence in host range, though the function of NS4 is not well characterized. Vpg (or NS5 in some sources), is a nonstructural protein that primes genome replication^77^.

Both novel E. dupreanum sapovirus 1 sequences were nearly identical to each other, but E. dupreanum sapovirus 1: accession OQ818319 is a partial genome and was missing the NS1/NS2 region of the genome, so it is possible some variation may exist in this region in the two sequences (Fig. 3F). The most similar virus, a Cameroonian *E. helvum*-hosted *Sapovirus*, hovered around 70% identity to the novel query sequences across most of the genome, with three drops in similarity at the border of Vpg and Pro-Pol, on the border of Pro-Pol and VP1, and in VP1 (Fig. 3F). The drops in identity were not as dramatic as those witnessed in certain regions for other novel Malagasy bat picornaviruses; in general, E. dupreanum sapovirus 1 displayed on average around 50% identity to previously characterized viruses in this clade (Fig. 3F). R. madagascariensis sapovirus 4 displayed the same dips in identity as E. dupreanum sapovirus 1. As observed in Malagasy bat picornaviruses (Fig. 3A-E), dips in identity across the genome corresponding to areas involving host interactions and immune responses could indicate that while *E. dupreanum* and *R. madagascariensis*-hosted sapoviruses were respectively most similar to African *E. helvum* and *R. aegypticu*s-hosted sapoviruses (range from ~ 67% to ~ 90% identity) (Supplementary Table 4), these novel viruses likely use different replication and immune evasion strategies.

### Coevolutionary analysis

#### Picornaviruses

**Fig. 4:**
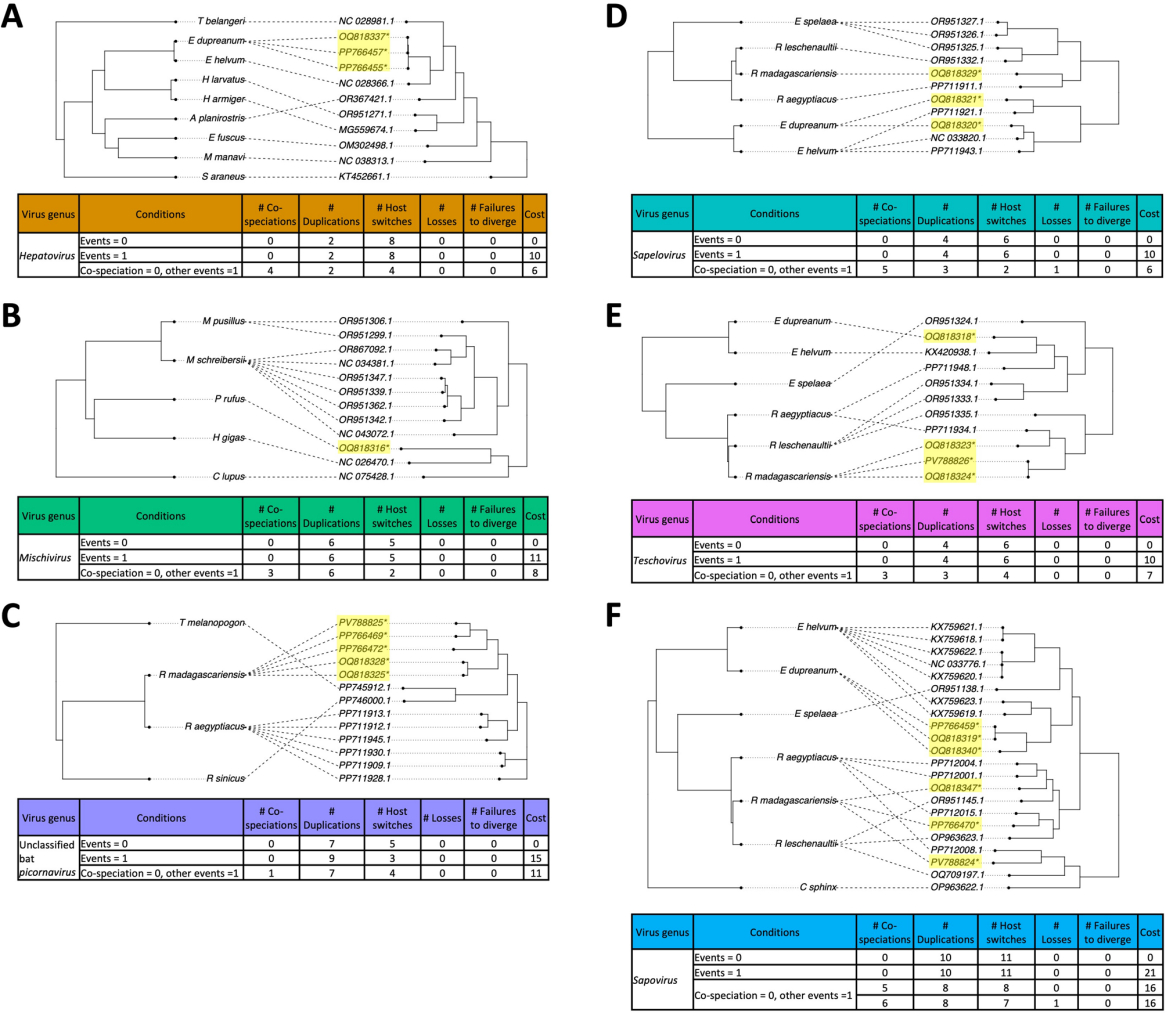
Tanglegrams and Jane^60^ co-evolutionary analysis output of host-virus relationships for (**A**) hepatoviruses, (**B**) mischiviruses, (**C**) unclassified bat picornaviruses, (**D**) sapeloviruses, (**E**) teschoviruses, and (**F**) sapoviruses. Novel viral sequences described in this study are highlighted in yellow and marked with an asterisk. The least costly evolutionary scenario is 0, which is further shown in Supplementary Fig. 6.

Through coevolutionary analysis, we determined that the least costly event for all virus-host co-phylogenies resulted in co-speciation followed by duplication and host switching as the most likely evolutionary scenario (Fig. 4A-E, Supplementary Fig. 6A-E). This evolutionary mechanism could help explain why our novel sequences have higher identity to those hosted by sister African bat species rather than those just from Madagascar. *Kunsagivirus* was excluded from this analysis due to lack of full genome reference sequences.

Ghanaian *E. helvum*-hosted *Hepatovirus* likely co-speciated with *E. dupreanum*-hosted hepatoviruses, with host-switching and duplication with the *Artibeus*-hosted *Hepatovirus*, further shown in the tanglegram (Fig. 4A, Supplementary Fig. 6A). As in hepatoviruses, there was no congruence between our novel *P. rufus*-hosted *Mischivirus* and *R. madagascariensis*-hosted unclassified bat picornaviruses with their most closely related viral clades (in *H. gigas* and *R. aegyptiacus*, respectively), again suggesting co-speciation of virus clades (Fig. 4B-C, Supplementary Fig. 6B-C). However, after the co-speciation event in Malagasy bat picornaviruses, multiple host-switching and duplication events from the novel viral clade likely led to the diversification of African *R. aegyptiacus*-hosted picornaviruses (Supplementary Fig. 6C). Coevolutionary analysis in sapeloviruses suggested congruence between those hosted by *R. madagascariensis* and *R. aegypticus*, indicating co-speciation (Fig. 4D, Supplementary Fig. 6D). There was incongruence in *Eidolon*-hosted sapeloviruses, with co-speciation between our novel Malagasy bat viruses and African *E. helvum*-hosted sequences (Fig. 4D, Supplementary Fig. 6D). A duplication and host-switch of E. dupreanum sapelovirus 1 likely led to the Cameroonian *E. helvum*-hosted lineage (Supplementary Fig. 6D). In teschoviruses, there was congruence between viral clades hosted by *E. dupreanum* and those hosted by *E. helvum*, with the same pattern observed in *R. madagascariensis* and *R. aegyptiacus*, indicating that co-speciation initially led to the diversification of these clades (Fig. 4E). After separate co-speciation events, R. madagascariensis teschovirus 1 likely duplicated and host-switched to a Kenyan *R. aegypticus* (Supplementary Fig. 6E). Further, host-switching and duplication from the Ugandan *R. aegypticus*-hosted *Teschovirus* lineage further diversified into the *Eidolon*-hosted *Teschovirus* lineage (Supplementary Fig. 6E).

#### Sapoviruses

As seen with picornaviruses, the least costly event for *Sapovirus* resulted in diversification by co-speciation followed by host-switching and duplication (Fig. 4F, Supplementary Fig.6F). Incongruence was observed amongst viral clades hosted by* R. madagascariensis*, *R. aegyptiacus*, and *R. leschenaultii* which has a high number of co-speciation events between *R. madagascariensis* and *R. leschenaultii* (Fig. 4F, Supplementary Fig. 6F). Further, host-switching and duplication events followed the initial *Rousettus* co-speciation, with two events stemming from sapoviruses hosted by Kenyan *R. aegyptiacus*, and two events stemming from sapoviruses hosted by Malagasy *R. madagascariensis* (Supplementary Fig. 6F). As seen in teschoviruses, there was congruence in sapoviruses hosted by *Eidolon*, indicating co-speciation between the viral lineages (Fig. 4F), with no evidence of host-switching and duplication between Cameroonian *E. helvum*-sapoviruses an *E. dupreanum*-hosted sapoviruses (Supplementary Fig. 6F).

### RDP4 recombination analysis

**Fig. 5:**
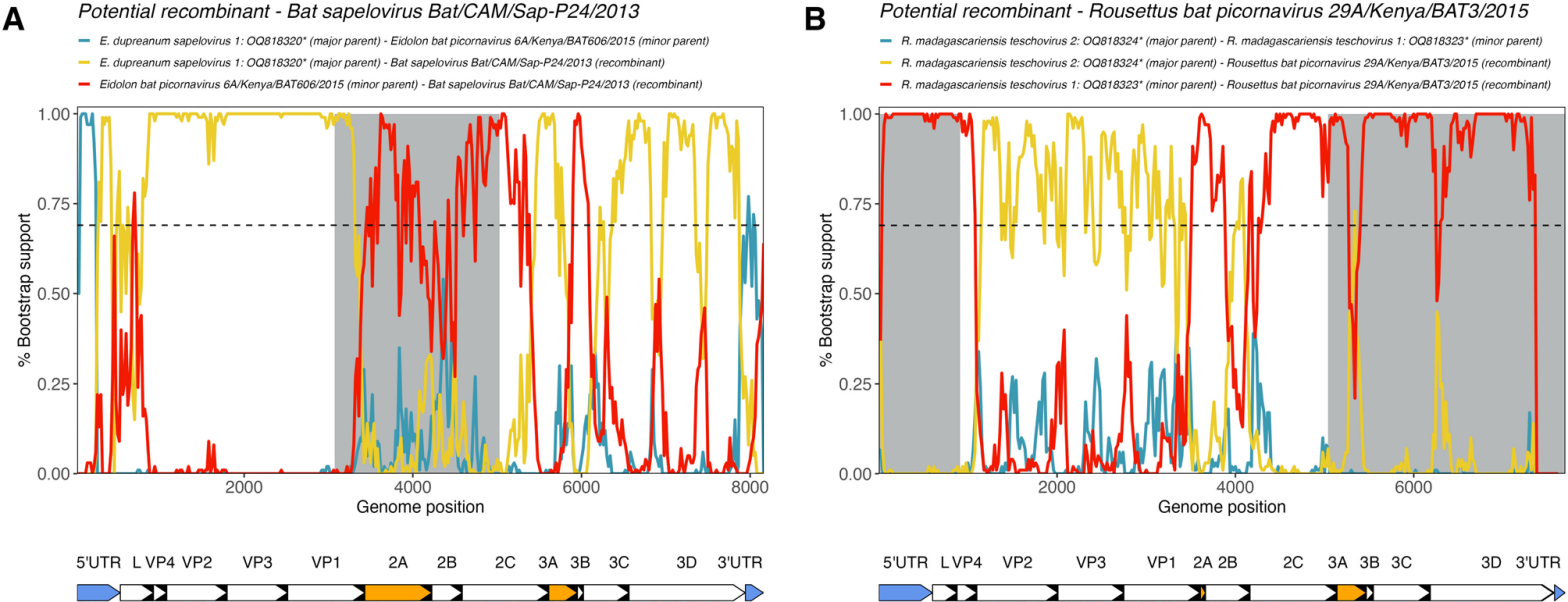
Bootscan plots computed in RDP4^66^ for potential recombinant sequences (**A**) Bat sapelovirus Bat/CAM/Sap-p24/2013: accession NC_033820 and (**B**) Rousettus bat picornavirus 29A/Kenya/BAT3/2015: accession PP711943. Line color corresponds to pairwise alignments between the potential recombinant sequence, major parental sequence, and the minor parental sequence. Asterisks denote novel sequences described in this study. Horizontal dashed line refers to a 70% cutoff bootstrap percentage, and grey bars indicate regions identified as significant areas of recombination (P < 0.05) across at least 5 analyses within RDP4^66^ (RDP, GENECONV, Bootscan, Maxchi, Chimaera, and 3Seq). Nucleotide bootscan plots were generated using a window size of 200 bp and a step size of 20 bp. Genome maps are below each plot, peptides in orange denote areas of interest for host interactions and immunogenicity, and blue peptides denote 5’ and 3’ UTRs. RDP4^66^ statistics are reported in Supplementary Table 9.

Recombination analysis performed on novel Malagasy picornaviruses indicated that there is evidence for genetic exchange with viruses hosted by African *E. helvum* and *R. aegypticus* (Fig. 5, Supplementary Table 9). Overall, the following genera displayed the strongest recombination signal: unclassified bat picornaviruses, *Hepatovirus*, *Sapelovirus*, and *Teschovirus* (Supplementary Table 9). No significant recombination pressure was observed in *Kunsagivirus*, *Kobuvirus*, *Mischivirus*, and *Sapovirus* (Supplementary Table 9). *Cardiovirus* was excluded from this analysis because of genome length.

Through RDP4^66^ analysis, R. madagascariensis picornavirus 4 was identified to be a possible recombinant with two Uguandan *R. aegypticus*-derived picornaviruses as major and minor parental sequences, respectively (Supplementary Fig. 7A, Supplementary Table 9). Bootstrap support indicated that there is some potential for the VP2 region to be under recombination pressure; however only the 3’- and 5’-UTR regions of the genome were significant (Supplementary Fig. 7A). *E. dupreanum*-hosted hepatoviruses were identified as possible minor parental sequences to recombinant *E. helvum*-hosted *Hepatovirus*; however, bootstrap support was too weak to indicate any other parental sources of genomic material (Supplementary Fig. 7B).

Within *Sapelovirus*, the strongest bootstrap support indicated that a Cameroonian *E. helvum*-hosted *Sapelovirus* was the likely recombinant sequence with genomic material from E. dupreanum sapelovirus 1 (major parental sequence) across the genome excepting the P2 region (2A, 2B, and 2C) and the 3A peptide where higher bootstrap support indicated that a Kenyan *E. helvum*-hosted *Sapelovirus* was the minor parental sequence (Fig. 5A). There was additional evidence that E. dupreanum sapelovirus 2 was a recombinant sequence with Kenyan *E. helvum*-hosted *Sapelovirus* as a minor parental sequence, with highest bootstrap support for this genomic history in the P2 (2A, 2B, 2C) region and lower bootstrap support in P1 region (VP4, VP2, VP3, and VP1) (Supplementary Fig. 7C). These findings are supported by the previously established co-evolutionary scenario for *Sapelovirus* (Fig. 4D, Supplementary Fig. 6D).

Evidence for recombination was highest overall within the *Teschovirus* genus (Fig. 5B and Supplementary Fig. 7D and 7E), where the most likely recombinant was identified as Kenyan *R. aegypticus*-hosted *Teschovirus*, with genomic material contributed from R. madagascariensis teschovirus 2 (major parental sequence) and R. madagascariensis teschovirus 1 (minor parental sequence), with higher support for the major parental sequence in the P1 region (VP4, VP2, VP3, and VP1) (Fig. 5B). Additional analysis with a consensus sequence of African *R. aegypticus*-hosted teschoviruses further supported the involvement of *R. madagascariensis*-hosted teschoviruses in recombination, but with lower bootstrap support across the whole genome (Supplementary Fig. 7D and 7E). Support for R. madagascariensis teschovirus 1 as a recombinant sequence with *R. aegypticus*-hosted *Teschovirus* clade as a minor parental sequence was highest in the 5’-UTR/3D region (Supplementary Fig. 7D). The opposite pattern was observed in the 3’-UTR in analysis of *R. aegypticus*-hosted *Teschovirus* clade as a recombinant sequence with R. madagascariensis teschovirus 1 as a major parental sequence (Supplementary Fig. 7E). VP1-VP3 peptides in picornaviruses are thought to play a role in generation of neutralizing antibodies^78^, and as previously mentioned, the P1-P2 junction, 3A/3B peptides, and 5’-UTR also play a role in determination of host range and immune response^19,57,76^. These findings are supported by the previously established co-evolutionary scenario for *Teschovirus* (Fig. 4E, Supplementary Fig. 6E).

## Discussion

Using mNGS data, we analyzed the diversity of picornaviruses and sapoviruses, finding evidence of high viral similarity between sequences from Malagasy fruit bats (*E. dupreanum* and *R. madagascariensis*) with those from their sister species (*E. helvum* and *R. aegypticus*, respectively) from mainland Africa, although it is possible the home ranges for these species simply represent the most intensely sampled localities to date.

Further, we find evidence of an evolutionary history of co-speciation followed by duplication and host-switching that likely led to the diversification of these viral genera in Malagasy fruit bats through co-evolutionary and recombination analyses. We have previously demonstrated potential cross-continental viral genetic exchange between African and Malagasy fruit bat-hosted nobecoviruses, so it is possible these picornaviruses and sapoviruses are diversifying in similar manners^40^. Multiple *Picornaviridae* and *Sapovirus* species were identified within the same sampling sessions at *E. dupreanum* and *R. madagascariensis* roost sites, suggesting that many of these diverse viruses shed simultaneously within a representative bat population, a pattern that has been reported before for fruit bats^79^. Indeed, viral diversity was significantly different between roost sites dominated by *E. dupreanum* versus *R. madagascariensis*, and sapeloviruses, teschoviruses, and sapoviruses all resolved into disparate host species-specific clades. *R. madagascariensis*-hosted picornaviruses, which displayed the lowest identity to previously-known sequences (~ 80% to known Ugandan/Kenyan *R. aegypticus*-hosted picornaviruses^69^), formed their own clade distinct from *Rousettus*-hosted viruses described elsewhere, which further separated into two separate clades corresponding to two different viral species.

Many of the novel sequences we identified in this analysis displayed reduced identity to previously-described sequences in genomic areas that determine host range and immune response (5’-UTR, 2A/2B peptides, and 3A/3B peptides in picornaviruses^19,57,76^, NS4 and VP1 peptides in *Sapovirus*). These regions of reduced similarity indicate that, despite relatedness to sister species-hosted viruses in Africa, Malagasy bat-hosted viruses could employ different replication and immune evasion mechanisms. Our recombination analysis did not identify the 3A region, which is highly diverse in picornaviruses and likely contributes to host range^57^, to be under recombination pressure. To our knowledge, recombination analysis has not been previously performed on any African bat picornaviruses and sapoviruses^29,30,69,80^. While no evidence for local recombination between the novel Malagasy viruses was found, analyses presented here suggest that a combination of duplications, host-switches, and recombination likely contributed to the diversification of these clades. This is supported by literature stating that *Picornaviridae* in general and their hosts diversify through host-switching mechanisms^28,38^, as do other viral families in Malagasy bats^43^. We did not find any evidence of heterologous virus recombination in this study, as previously demonstrated between a fusogenic *Orthoreovirus* and a *Nobecovirus*^81^, although some members of *Picornaviridae *(particularly *Enterovirus*^82^) have been shown to recombine on an intra-species level frequently, resulting in mosaic genomes.

Of note, *E. dupreanum* and *R. madagascariensis* are known to co-roost in caves in our system^40^, while *P. rufus* is tree-dwelling. Host proximity plays a key role in viral dispersal, exchange, and diversification. Cave co-roosting has been previously shown to support recombination and diversification in bat coronavirus systems^83^. As *E. dupreanum* and *R. madagascariensis* are also known to co-roost with insectivorous bats on occasion^84^, additional screening for *Picornavirales* in more Malagasy bat species may further support evidence for host-switching between co-roosting bat species on a local scale. It is likely that the tree-roosting behavior of *P. rufus* contributes to the lack of shared viruses with other fruit bats observed in this study – as only one *Picornavirales* genome was identified in *P. rufus*, representing a *Mischivirus* not found in the other two species. The divergent evolutionary history of the *Pteropus* genus, as compared to the more proximal *Eidolon* and *Rousettus* clades^85^, also likely contributes to the lack of viral sharing between these disparate taxa.

Our study has several limitations. Chiefly, in our co-evolutionary analysis in Jane, only three cost scenarios were tested in concordance with prior work investigating the evolutionary history of bat picornaviruses^28^. Thus, our analysis evaluates rough hypotheses of co-speciation vs. complex speciation only – without considering alternative pathways that explicitly favor other event types (e.g. host switching) or more strongly favor any event type (e.g. by setting other event costs to values > 1). As few prior studies have used Jane to investigate bat virus co-evolutionary histories^28,86^, we felt that these more extreme scenarios could not be justified as biologically plausible; however, we acknowledge that this biased our analysis to favor coevolutionary models of host-parasite speciation. In addition, while parameter presets for population size and generation time are known to impact downstream inference in Jane^60^, we reported results for default values only, after identifying largely comparable outcomes across a range of recommended values^60^. It remains possible, however, that even smaller population sizes or generation numbers could yield more variable outcomes. Finally, we also adhered to default parameter values in recombination analysis conducted in RDP4. While variable inputs to RDP4 can modulate downstream results, we only accepted recombination signals significant using at least five of six recombination detection methods used in this software, making us confident in the accuracy of our inference. While undersampling potential parent lineages is always an issue in recombination analysis, RDP4 alleviates some of these biases by allowing for the possibility that an unknown parent may be a more likely donor than those included in the alignment^66^.

In Madagascar and some areas of mainland Africa, humans hunt bats for food^87–89^. While no bat-borne zoonosis has been linked to this practice in Madagascar, undiagnosed fevers are common, and it is possible that bat virus zoonoses may be occurring undetected^90,91^. Enteric viruses described from Cameroonian hunters demonstrated a high diversity of *Picornavirales*, including some sequences which share evolutionary ancestry with bat- or other animal-hosted viruses^80^. Nonetheless, human-hosted *Picornavirales* in Cameroon segregate phylogenetically from animal viruses, suggesting that, while zoonosis may be possible in this clade, these cross-species emergence events are relatively rare^80^. We found that the closest relatives of our sequences were in sister bat species, when available for comparison. In these sister species, it does appear that these enteric viruses circulate within the bat populations at higher levels than other co-circulating viruses, particularly within the family *Picornaviridae*^69^, and resulting in their detection in over half of the available pools for sequencing (76% for *Picornaviridae*^30^ and 56% for *Sapovirus*^29^). The bat-hosted viral clades identified in this study were not closely related to any human-hosted sequences (*Cardiovirus*, *Hepatovirus*, *Kobuvirus*, *Sapovirus*). Indeed, discrete host-species relationships appear to drive most of the observed diversification within the *Picornavirales* clade. While we lack *Picornavirales* sequences from humans in Madagascar to explicitly evaluate the potential for zoonosis, the bat sequences described here group with other animal (particularly bat)-derived viruses from related host species^69^. Increased sampling of additional hosts, including humans with high bat contact (e.g. bat hunters) or farm animals with high human contact, would be beneficial to untangling the potential zoonotic risk posed by Malagasy bat-borne *Picornavirales*. As recombination events can precede more dramatic host-switches, including zoonoses, understanding of these processes within diverse viral clades is critical to assessing potential zoonotic risk.

## Supplementary Information

Below is the link to the electronic supplementary material.

Supplementary Material 1

Supplementary Material 2

## Data Availability

All full and partial length genome sequences were submitted to GenBank (https://www.ncbi.nlm.nih.gov/genbank/) and assigned accession numbers OQ818316-OQ818318, OQ818320-OQ818324, OQ818328, OQ818329, PP766456, PP766459, PP766469 (full-length genomes), and OQ818319, OQ818325, OQ818337, OQ818340, OQ818342-OQ818348, PP766449-PP766455, PP766457, PP766458, PP766460-PP766468, PP766470-PP766477 (partial-length genomes). Detailed descriptions of analyses done in this paper, including scripts used to generate figures, are available on our GitHub (https://github.com/brooklabteam/Kettenburg_mada-bat-picornavirus).
