# Supplementary material for: Co-speciation and host-switching drives diversity of picornaviruses and sapoviruses in Malagasy fruit bats
Source: Sci Rep. 2026 Jan 23;16:6583. doi: 10.1038/s41598-025-34969-2 (PMC12914028; doi:10.1038/s41598-025-34969-2)
Supplement: Supplementary file 1 — Supplementary Material 1 [file 41598_2025_34969_MOESM1_ESM.pdf]

SUPPLEMENTARY FIGURES

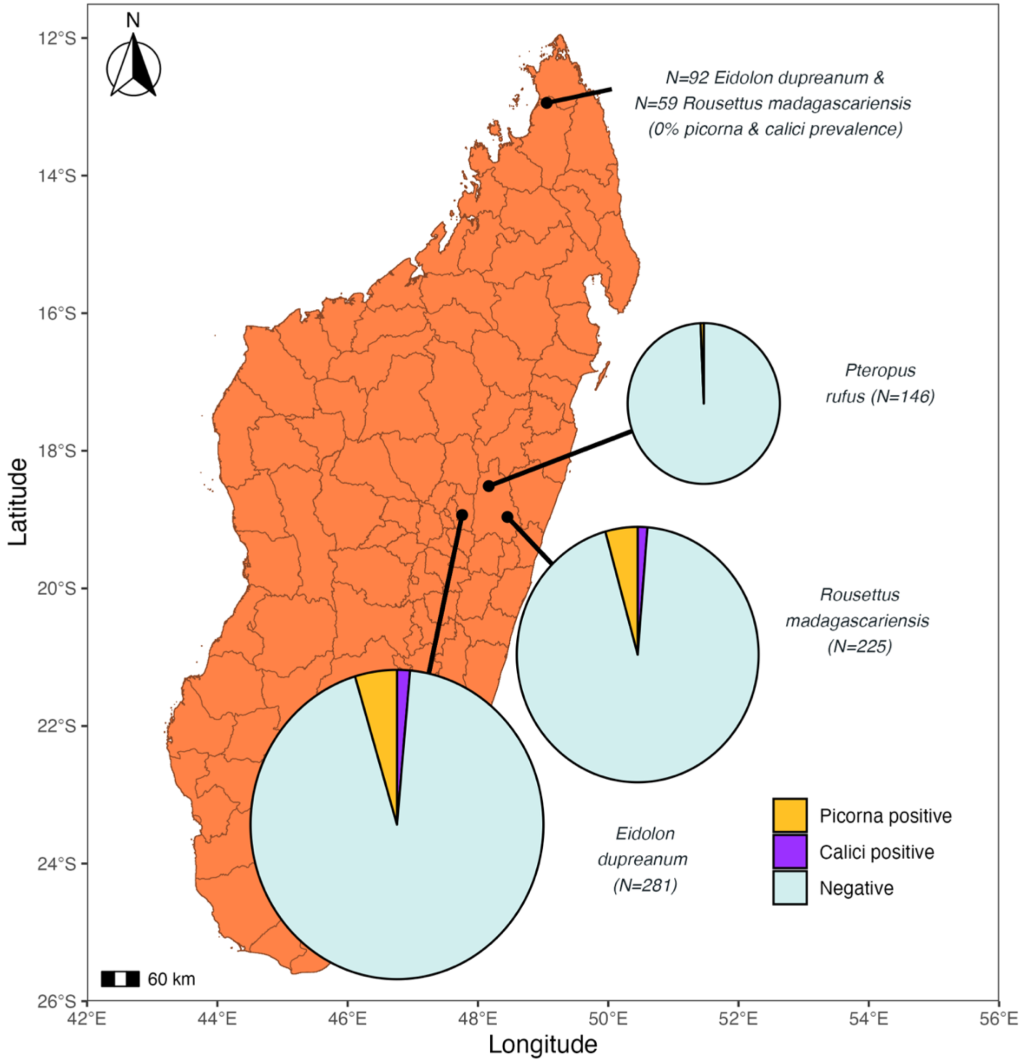

Supplementary figure 1: Map of sampling sites for *Eidolon dupreanum, Pteropus rufus*, and *Rousettus madagascariensis* at sites in Districts of Ambilobe (*E. dupreanum* and *R. madagascariensis*: Ankarana caves), Moramanga (*P. rufus:* Ambakoana roost and *R. madagascariensis*: Maromizaha cave), and Manjakandriana (*E. dupreanum*: Angavobe/Angavokely caves), Madagascar. Pie charts show *Picornaviridae* (yellow) and *Caliciviridae* (orange) positive bats by site. Pie chart size corresponds to sample size on a log10 scale. Map and pie charts were created in RStudio using the packages 'maptools', 'sf', and 'scatterpie'.

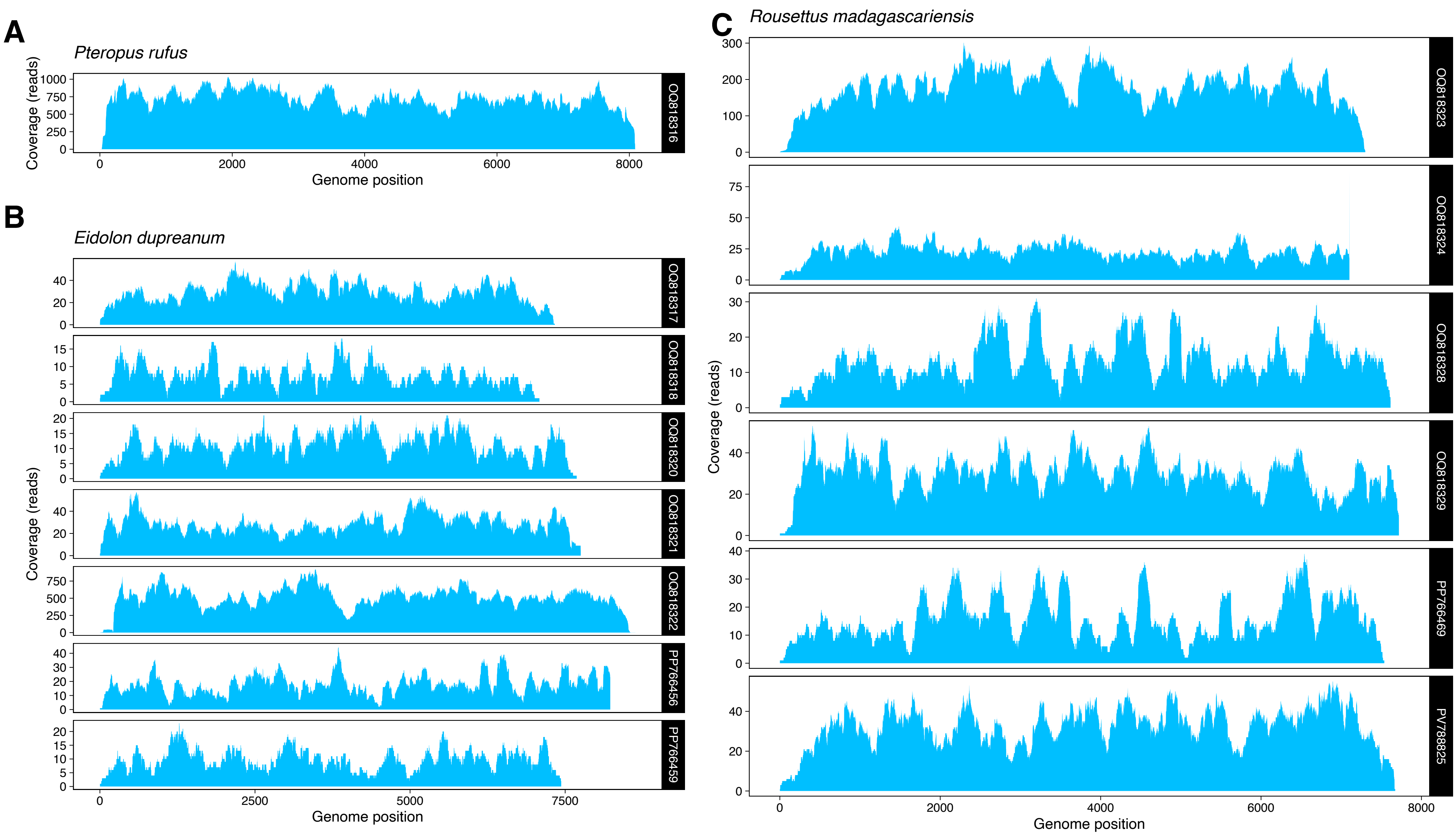

Supplementary figure 2: Read depth and coverage after deduplication by CD-HIT[1] for full-length contigs assembled in CZID recovered from (A) *P. rufus*, (B) *E. dupreanum*, and (C) *R. madagascariensis*. Contig depth is shown in the plots as raw coverage (number of reads mapped to genome at each nucleotide position) recovered for each full genome sequence.

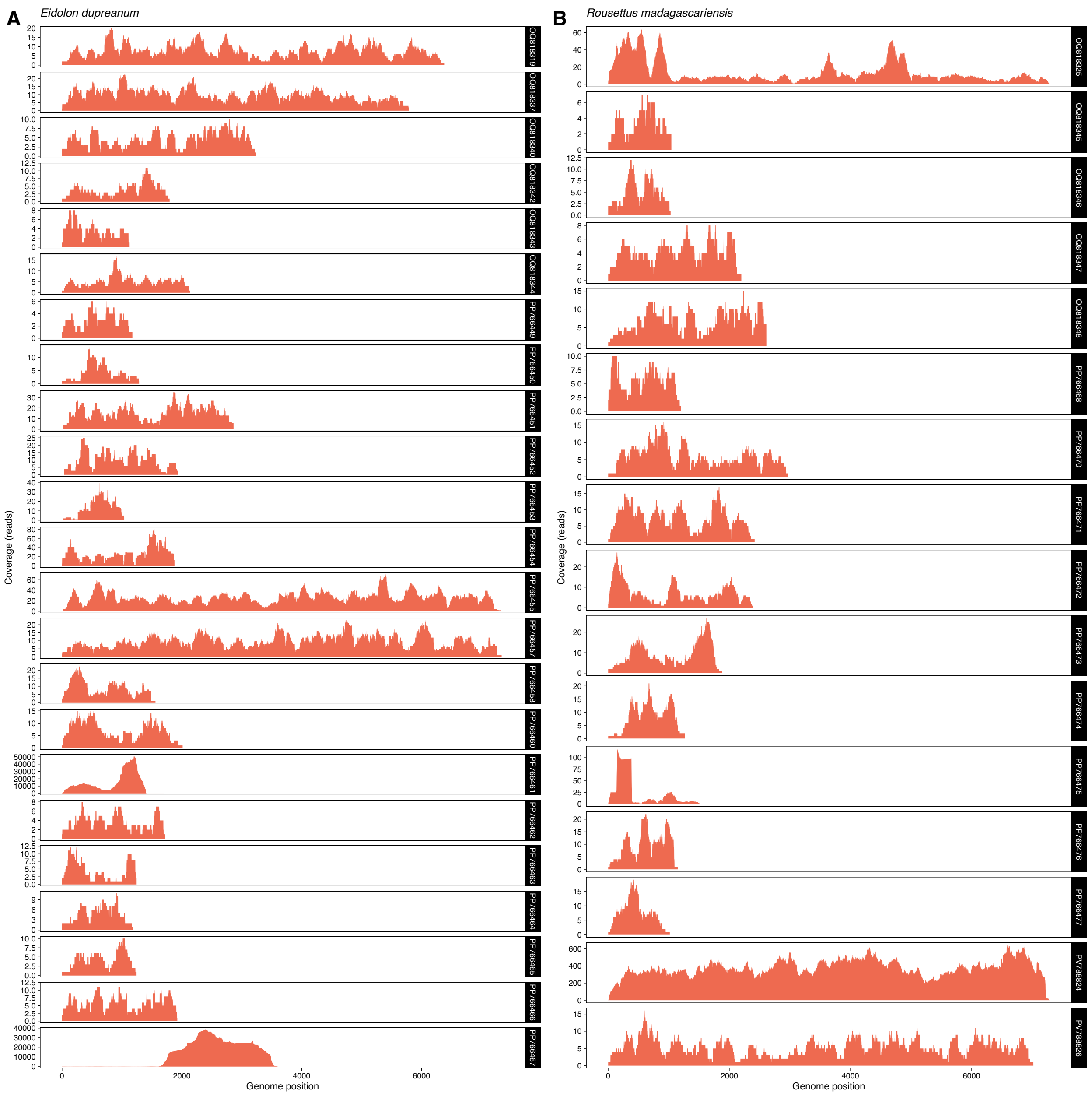

Supplementary figure 3: Read depth and coverage after deduplication by CD-HIT[1] for partial-length contigs assembled in CZID recovered from (A) *E. dupreanum* and (B) *R. madagascariensis*. Contig depth is shown in the plots as raw coverage (number of reads mapped to genome at each nucleotide position) recovered for each partial genome sequence.

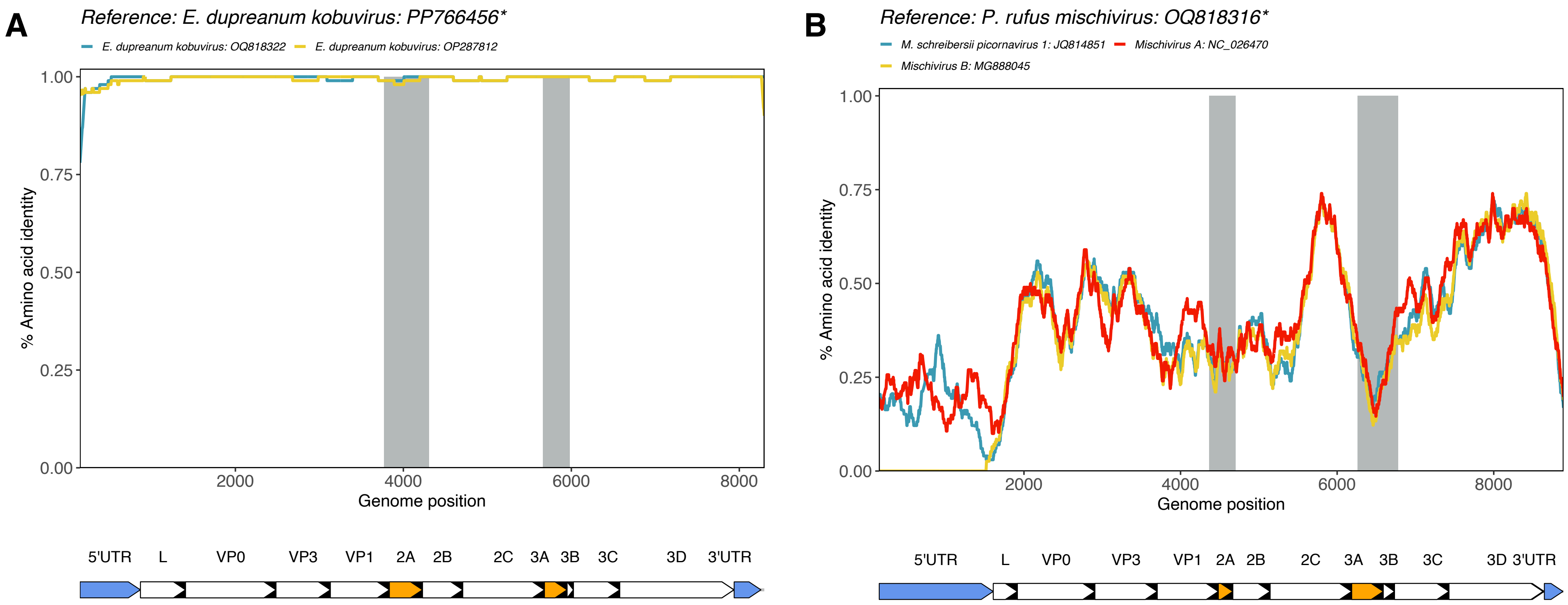

Supplementary figure 4: Amino acid similarity computed in PySimPlot[2] for novel full-length sequences. Similarity analyses with query sequence (A) E. dupreanum kobuvirus: accession PP766456 and (B) P. rufus mischivirus: accession OQ818316 against similar sequences identified from BLAST[3] and other matched novel sequences within the same genus. Asterisks denote novel sequences described in this study. Line color corresponds to different virus sequences, with annotated regions of the genome below each plot. Peptides in orange and corresponding grey shaded areas denote areas of interest for host interactions and immunogenicity, and blue peptides denote 5' and 3' UTRs.

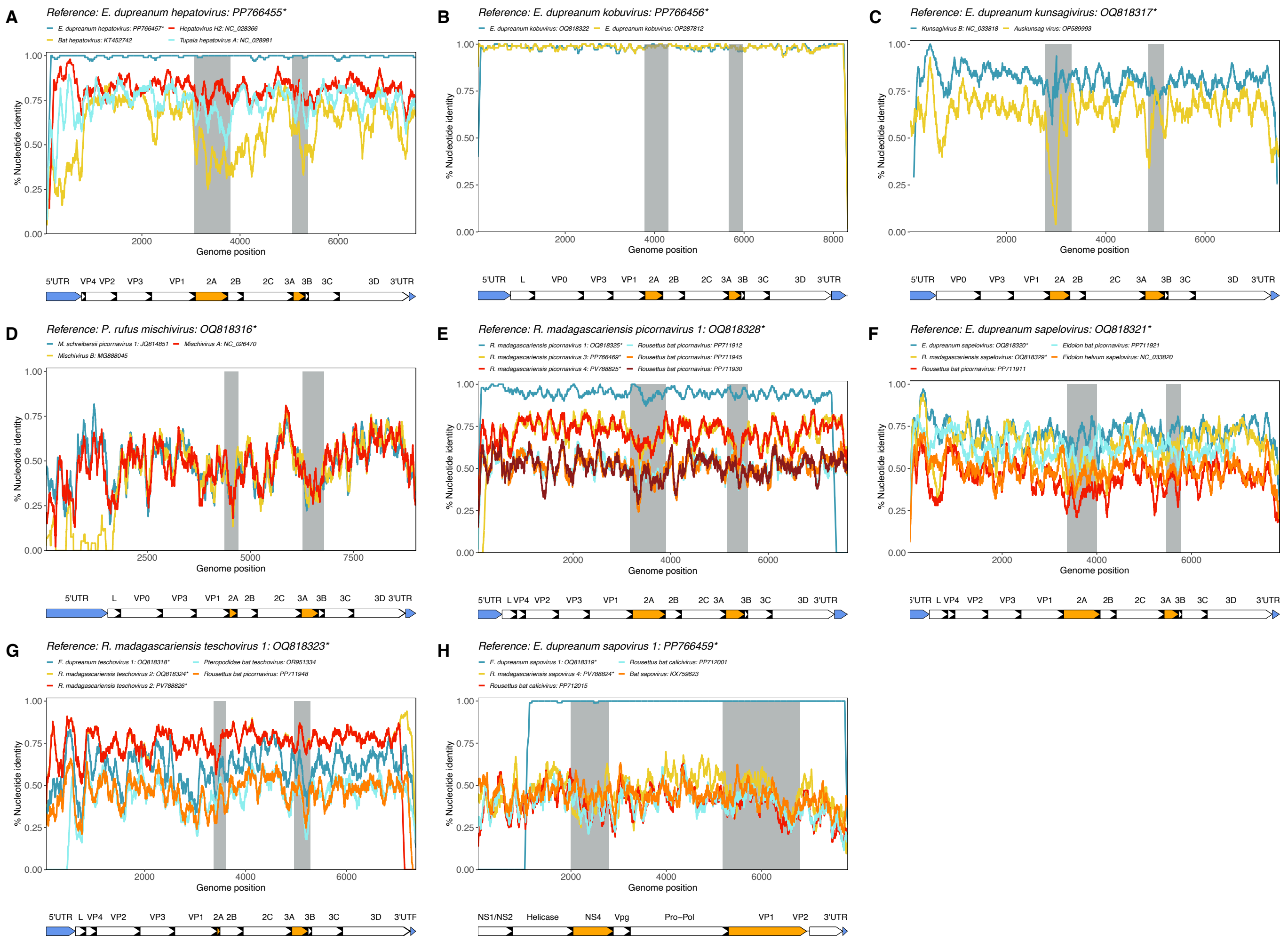

Supplementary figure 5: Nucleotide similarity computed in PySimPlot[2] for novel full-length sequences. Similarity analyses with query sequence (A) E. dupreanum hepatovirus: accession PP766455, (B) E. dupreanum kobuvirus: accession PP766456, (C) E. dupreanum kunsagivirus: accession OQ818317, (D) P. rufus mischivirus: accession OQ818316, (E) R. madagascariensis picornavirus 1: accession OQ818325, (F) E. dupreanum sapelovirus: accession OQ818321, (G) R. madagascariensis teschovirus 1: accession OQ818323, and (H) E. dupreanum sapovirus 1: accession PP766459 against similar sequences identified from BLAST[3] and other matched novel sequences within the same genus. Asterisks denote novel sequences described in this study. Line color corresponds to different virus sequences, with annotated regions of the genome below each plot. Peptides in orange and corresponding grey shaded areas denote areas of interest for host interactions and immunogenicity, and blue peptides denote 5' and 3' UTRs. Plots were generated with a window size of 200bp and a step size of 20bp.

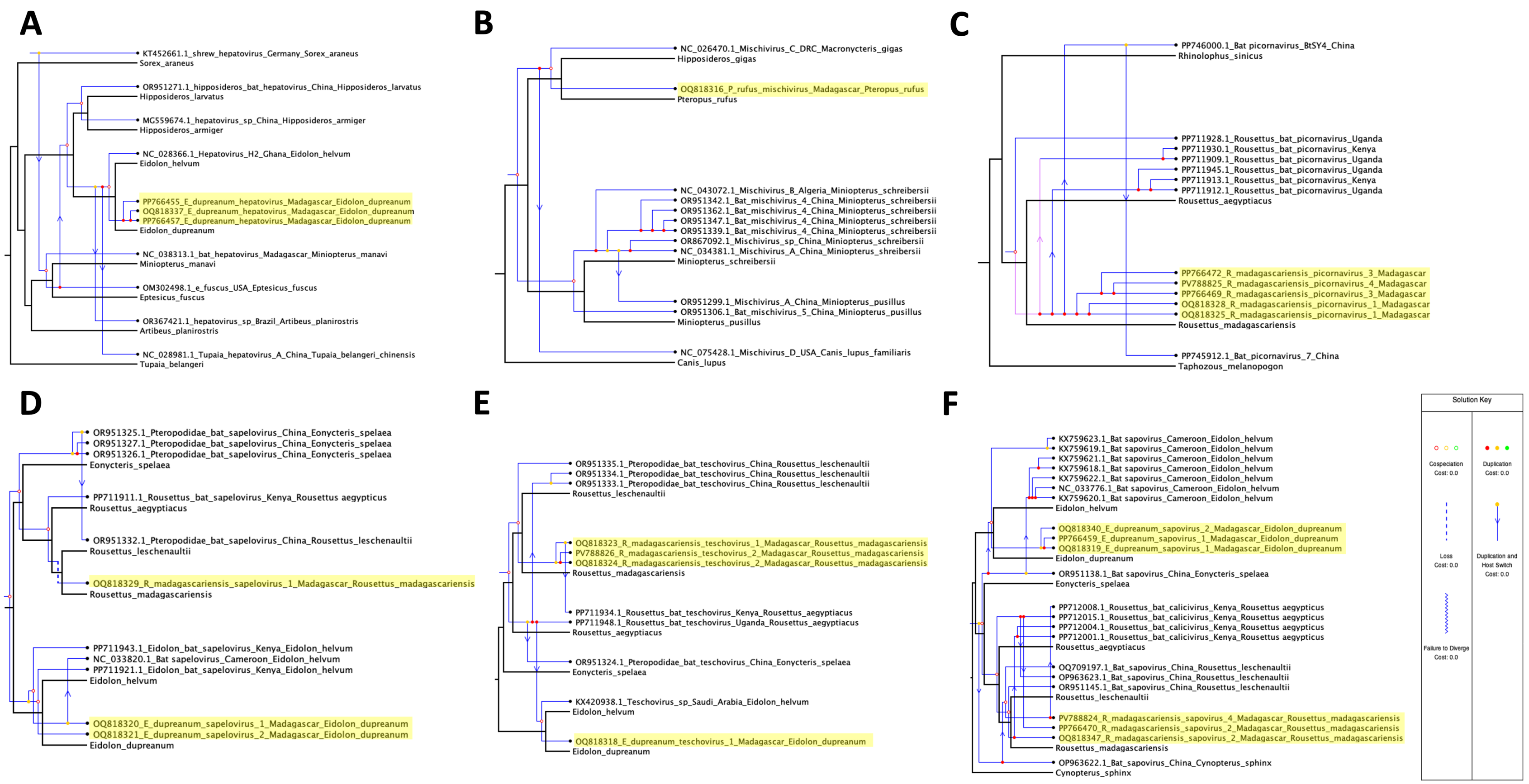

Supplementary figure 6: Co-phylogeny of the least costly evolutionary scenario for (A) henipaviruses, (B) mischiviruses, (C) unclassified bat picornaviruses, (D) sapeloviruses, (E) teschoviruses, and (F) sapoviruses and their hosts. Black phylogeny represents the host, and blue branches represent the viruses. Open circles represent co-speciation events, closed circles represent duplication events, and closed circles with a blue arrow represent a duplication and host switch. Novel viral sequences described in this study are highlighted in yellow.

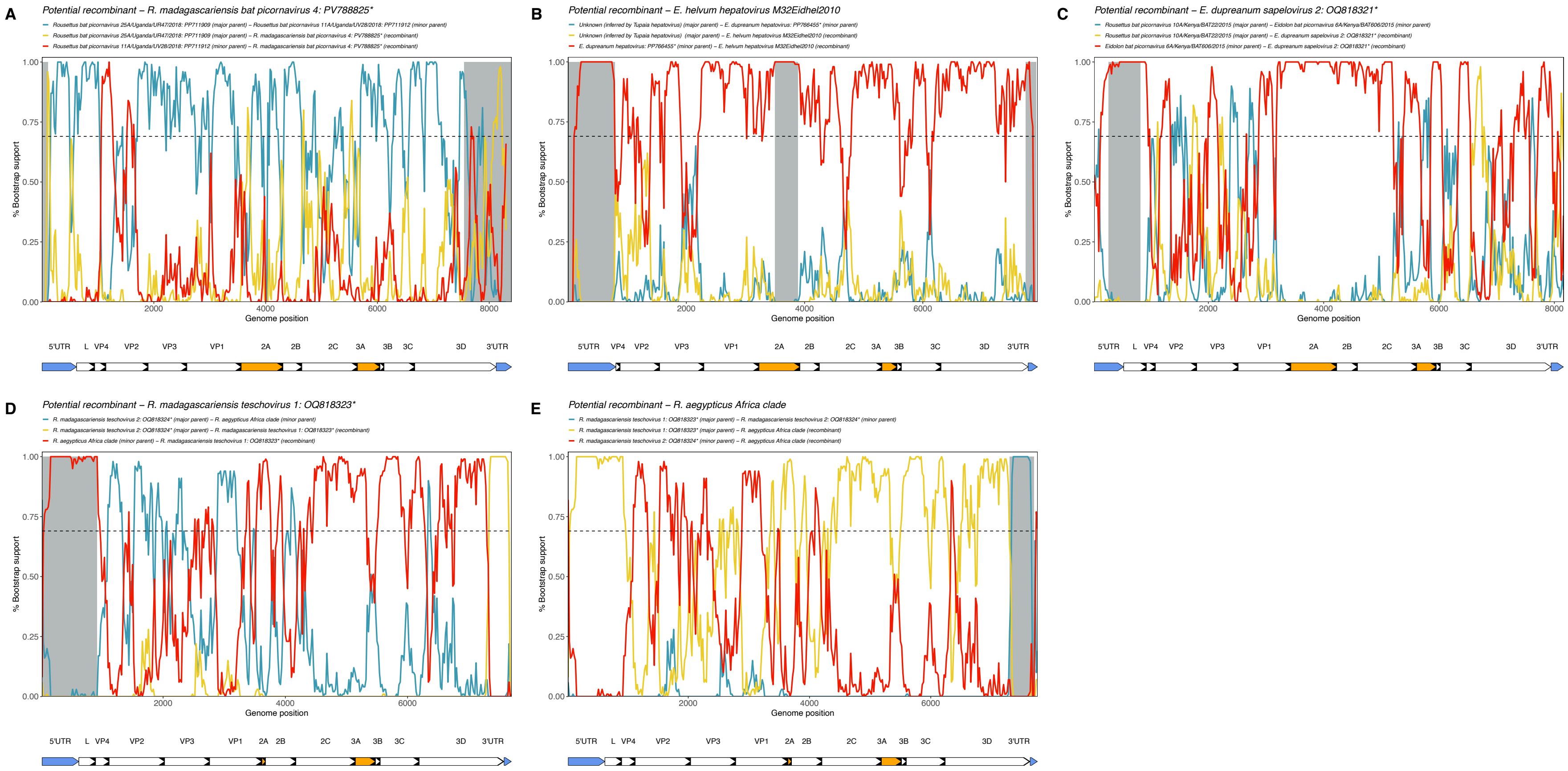

Supplementary figure 7: Bootscan plots computed in RDP4[4] for potential recombinant sequences (A) R. madagascariensis picornavirus 4: PV788825, (B) E. helvum hepatovirus M32Eidhel2010: accession NC_028366, (C) E. dupreanum sapelovirus 2: accession OQ818321, (D) R. madagascariensis teschovirus 1: OQ818323, and (E) R. aegypticus Africa clade (accessions: PP711948 and PP711934). Line color corresponds to pairwise alignments between the potential recombinant sequence, major parental sequence, and the minor parental sequence. Asterisks denote novel sequences described in this study. Horizontal dashed line refers to a 70% cutoff bootstrap percentage, and grey bars indicate regions identified as significant areas of recombination (P<0.05) across at least 5 analyses within RDP4[4] (RDP, GENECONV, Bootscan, Maxchi, Chimaera, and 3Seq). Nucleotide bootscan plots were generated using a window size of 200bp and a step size of 20bp. Genome maps are below each plot, peptides in orange denote areas of interest for host interactions and immunogenicity, and blue peptides denote 5' and 3' UTRs. Full RDP4[4] statistics are in **Supplementary table 9**.

SUPPLEMENTARY TABLES – See separate excel file for tables for corresponding table figure captions listed below

Supplementary Table 1: Summary information of post-sequencing CZID results.

Supplementary table 2: Summary information of phylogenies presented in **Figure 1B** and **2**.

Supplementary table 3: Summary table of BLASTx[3] and BLASTn[3] results of novel full and partial-length picornaviruses recovered from mNGS. Bold denotes full-length sequences.

Supplementary table 4: Summary table of BLASTx[3] and BLASTn[3] results of novel full and partial-length sapoviruses recovered from mNGS. Bold denotes full-length sequences.

Supplementary table 5: Peptide cleavage sites for full and partial-length picornaviruses described in this study. Bold denotes full-length sequences.

Supplementary table 6: Conserved motifs in novel picornaviruses from Madagascar fruit bats. Bold denotes full-length sequences. Dashes indicate that the sequence recovered does not include that motif due to length. Absent motifs are otherwise noted.

Supplementary table 7: Peptide cleavage sites for full and partial-length sapoviruses described in this study. Bold denotes full-length sequences.

Supplementary table 8: Conserved motifs in novel *Sapovirus* sequences from Madagascar fruit bats. Bold denotes full-length sequences. Dashes indicate that the sequence recovered does not include that motif due to length. Absent motifs are otherwise noted.

Supplementary table 9: RDP4[4] output of comparisons involving novel sequences as either the recombinant sequence or a major/minor parental sequence. Unknown followed by an accession number indicates that RDP4[4] used that named sequence to infer an unknown parental sequence. NS denotes non-significance in that analysis. Recombination events are accepted if at least 4 tests are significant. Recombinant sequences with highest support are highlighted in red (5-6 significant tests). Novel sequences identified in this study are in bold. Particular lineages are consensus sequences of aligned phylogenetic clade members and include: Rhinolophus bat picornavirus BtSY4 clade (accessions OP963617 and PP746000), *Shanbavirus* A clade (accessions KJ641687, KJ641690, KJ641699, and KJ641693), *R. aegypticus* African picornavirus clade (accessions PP711912, PP711945, PP711913, PP711930, PP711928, and PP711909), *H. larvatus* Chinese *Hepatovirus* clade (accessions OR951274, OR951275, OR867086,

OR951271), *Rattus tanezumi* Chinese *Kobuvirus* clade (accessions OM069746 and OM069755), *Rhinolophus* Chinese *Kobuvirus* clade (accessions OR951245 and OR951365), Rodent Chinese *Kunsagivirus* clade (accessions OQ716009 and OQ716008), *Eonycteris* Chinese *Sapelovirus* clade (accessions OR951327, OR951326, OR951325, and OR951332), *E. helvum* Cameroonian *Sapovirus* clade (accessions KX759619, KX759623, KX759621, NC_033776, and KX759622), *R. aegypticus* Kenyan *Sapovirus* clade (accessions PP712001, PP712004, PP712008, and PP712015), *R. aegypticus* east African *Teschovirus* clade (accessions PP711948 and PP711934), and *Rousettus* Chinese *Teschovirus* clade (accessions OR951335, OR951333, and OR951334).

SUPPLEMENTARY REFERENCES:

1. Fu, L., Niu, B., Zhu, Z., Wu, S. & Li, W. CD-HIT: accelerated for clustering the next-generation sequencing data. *Bioinformatics* **28**, 3150–3152 (2012).
2. Davies. PySimPlot. *GitHub* https://github.com/jonathanrd/PySimPlot.
3. BLAST® Command Line Applications User Manual.
4. Martin, D. P., Murrell, B., Golden, M., Khoosal, A. & Muhire, B. RDP4: Detection and analysis of recombination patterns in virus genomes. *Virus Evol.* **1**, vev003 (2015).
5. Conow, C., Fielder, D., Ovadia, Y. & Libeskind-Hadas, R. Jane: a new tool for the cophylogeny reconstruction problem. *Algorithms Mol. Biol.* **5**, 16 (2010).
