# Supplementary material for: Co-speciation and host-switching drives diversity of picornaviruses and sapoviruses in Malagasy fruit bats
Source: Sci Rep. 2026 Jan 23;16:6583. doi: 10.1038/s41598-025-34969-2 (PMC12914028; doi:10.1038/s41598-025-34969-2)
Supplement: Supplementary file 2 — Supplementary Material 2 [file 41598_2025_34969_MOESM2_ESM.pdf]

Supplementary Table 1: Summary information of post-sequencing CZID results.

Sample group	SampleID	Total sample reads	ERCC reads	ERCC linear	Reads passing filters	Pass QC	Virus family	NT/NR reads per virus family
NIH	Plate45_C6	72,382,862	8 (0%)	no (not used)	5,522,502 (7.63%)	93.48%	*Picornaviridae*	137/184
NIH	Plate114_B9	43,982,106	1,098 (0%)	no (not used)	5,662,504 (12.87%)	91.62%	*Picornaviridae*	647/710
NIH	Plate114_B7	70,745,704	1,048 (0.00%)	no (not used)	10,423,546 (14.73%)	92.20%	*Picornaviridae*	896/990
NIH	KEL168	53,993,066	7,532 (0.01%)	no (not used)	5,310,876 (9.84%)	91.45%	*Caliciviridae*	14/502
NIH	KEL273	27,464,336	80 (0.00%)	no (not used)	4,843,098 (17.63%)	92.94%	*Picornaviridae*	54/178
NIH	KEL298	28,479,620	110 (0.00%)	no (not used)	2,548,376 (8.95%)	94.18%	*Picornaviridae*	127/126
NIH	Plate_42_C2	13,746,818	8 (0.00%)	no (not used)	991,810 (7.21%)	85.35%	*Picornaviridae*	4,995/5,018
NIH	Plate45_D6	87,672,094	28 (0.00%)	no (not used)	7,936,500 (9.05%)	93.53%	*Picornaviridae*	1,385/1,395
NIH	Plate_25_C8	46,323,502	58 (0.00%)	no (not used)	102,658 (0.22%)	85.87%	*Picornaviridae*	1,041/1,045
NIH	Plate_32_A10	145,168,640	6,120 (0.00%)	no (not used)	3,926,360 (2.70%)	87.73%	*Picornaviridae*	897/1,179
NIH	Plate_32_A10	145,168,640	6,120 (0.00%)	no (not used)	3,926,360 (2.70%)	87.73%	*Caliciviridae*	17/1,466
CZB	RR034B_029	62,438,032	2,828,800 (4.53%)	yes	1,504,324 (2.41%)	76.83%	*Picornaviridae*	0/39,118
CZB	RR034B_079	117,276,390	389,872 (0.33%)	yes	3,682,276 (3.14%)	85.47%	*Picornaviridae*	1,976/2,149
CZB	RR034B_094	32,858,460	57,448 (0.17%)	yes	4,811,803 (14.64%)	94.26%	*Picornaviridae*	538/560
CZB	RR034B_096	44,295,222	15,746 (0.04%)	yes	3,954,499 (8.93%)	92.30%	*Caliciviridae*	11/547
CZB	RR034B_130	33,895,162	83,360 (0.25%)	yes	8,802,880 (25.97%)	95.62%	*Caliciviridae*	54/106
CZB	RR034B_137	88,959,312	85,546 (0.10%)	yes	2,829,785 (3.18%)	79.94%	*Caliciviridae*	114/250
CZB	RR034B_163	46,343,504	509,994 (1.10%)	yes	7,339,609 (15.84%)	94.28%	*Picornaviridae*	606/620
CZB	RR034B_189	32,464,964	344,190 (1.06%)	yes	9,812,766 (30.23%)	95.50%	*Picornaviridae*	1,559/1,577
CZB	RR034B_190	39,315,598	50,536 (0.13%)	yes	1,425,068 (3.62%)	78.56%	*Picornaviridae*	169/209
CZB	RR034B_233	27,847,184	307,156 (1.10%)	yes	1,287,958 (4.63%)	87.13%	*Caliciviridae*	0/80
CZB	RR034B_239	53,902,938	13,182,072 (24.46%	yes	3,521,436 (6.53%)	91.75%	*Caliciviridae*	234/18,973
CZB	RR034B_242	63,545,254	689,362 (1.08%)	yes	8,328,483 (13.11%)	93.58%	*Picornaviridae*	1,709/1,752
CZB	RR034B_244	95,323,590	235,386 (0.25%)	yes	8,586,524 (9.01%)	91.52%	*Picornaviridae*	8,920/8,936
CZB	RR034B_248	16,855,124	30,694 (0.18%)	yes	3,193,670 (18.95%)	93.65%	*Picornaviridae*	26/83
CZB	RR034B_259	18,588,814	84,804 (0.46%)	yes	2,564,766 (13.80%)	93.58%	*Picornaviridae*	3/1,108
CZB	RR034B_268	31,773,930	153,368 (0.48%)	yes	6,475,500 (20.38%)	94.67%	*Picornaviridae*	408/1
CZB	RR034B_271	22,420,522	354,782 (1.58%)	yes	859,216 (3.83%)	81.04%	*Caliciviridae*	4/215
CZB	RR034B_281	43,437,908	239,212 (0.55%)	yes	3,106,408 (7.15%)	90.00%	*Picornaviridae*	1/263
CZB	RR034B_288	150,000,000	96,442 (0.06%)	yes	9,193,416 (6.13%)	90.95%	*Picornaviridae*	701/14
CZB	RR034B_290	20,903,666	228,622 (1.09%)	yes	1,239,754 (5.93%)	88.03%	*Picornaviridae*	1,584/1,608
CZB	RR034B_393	16,151,594	282,268 (1.75%)	yes	4,092,698 (25.34%)	95.00%	*Picornaviridae*	188/295
CZB	RR034B_412	15,266,680	129,268 (0.85%)	yes	763,626 (5.00%)	95.22%	*Picornaviridae*	242/242
CZB	RR034B_481	22,122,724	2,895,148 (13.09%)	yes	1,989,950 (9.00%)	89.78%	*Caliciviridae*	0/335

Supplementary Table 2: Summary information of phylogenies presented in **Figure 1A and 2.**

Phylogeny	Figure	Alignment file	# Novel seq	# Reference seq	Region	TaxID references	Overlap length (bp)	Best model
Summary polymerase	1A	polymerase_summary_align_trim	22	274	Polymerase	All taxid listed below	~2900	TIM2+F+R10
Cardiovirus	2A	cardio_all_align	1	75	Full genome	12103, 434308	~3600	TIM2+F+R10
Hepatovirus	2B	hepato_all_align	3	126	Full genome	12091, 1714618	~4500	GTR+F+I+G4
Kobuvirus	2C	kobu_all_align	3	180	Full genome	194960, 655314	~1090	GTR+F+R10
Kunsagivirus	2D	kunsagi_all_align	1	4	Full genome	1755589	~6700	TIM2e+R2
Mischivirus	2E	mischi_full_align	1	16	Full genome	1511778	~3200	GTR+F+R4
Unclassified bat *picornavirus*	2F	bat_picorna_all_align	5	35	Full genome	2169640, 2788549, 1281456	~1700	GTR+F+R5
Sapelovirus	2G	sapelo_all_align	3	57	Full genome	686982, 1073966	~7200	TIM2+F+R10
Teschovirus	2H	tescho_full_align	4	35	Full genome	118139, 2004714	~3200	TIM2+F+R9
Sapovirus	2I	sapo_all_align	6	217	Full genome	95341, 371833	~1800	GTR+F+R10

Supplementary Table 3: Summary table of BLASTx and BLASTn results of novel full and partial-length picornaviruses recovered from mNGS. Bold denotes full-length sequences.

Accession	Virus	BLASTx host species	BLASTx coverage (%)	BLASTx identity (%)	BLASTx accession	BLASTn host species	BLASTn coverage (%)	BLASTn identity (%)	BLASTn accession
PP766467	E. dupreanum cardiovirus	*Pongo*	91	95.42	QMI58083.1	*Rattus norvegicus*	97	83.04	DQ835185.2
OQ818337	E. dupreanum hepatovirus	*Eidolon helvum*	87	94.75	YP_009179216.1	*Eidolon helvum*	97	82.4	NC_028366.1
PP766455	E. dupreanum hepatovirus	*Eidolon helvum*	91	94.3	YP_009179216.1	*Eidolon helvum*	78	99.54	NC_028366.1
PP766457	E. dupreanum hepatovirus	*Eidolon helvum*	90	94.26	YP_009179216.1	*Eidolon helvum*	97	82.16	NC_028366.1
PP766458	E. dupreanum hepatovirus	*Eidolon helvum*	99	92.68	YP_009179216.1	*Eidolon helvum*	99	81.53	NC_028366.1
PP766449	E. dupreanum kobuvirus	*Eidolon helvum*	69	99.33	AGL97808.1	*Eidolon dupreanum*	100	98.99	OP287812.1
PP766451	E. dupreanum kobuvirus	*Eidolon dupreanum*	99	99.47	WBP49885.1	*Eidolon dupreanum*	99	98.48	OP287812.1
PP766452	E. dupreanum kobuvirus	*Eidolon dupreanum*	99	99.31	WBP49885.1	*Eidolon dupreanum*	100	97.88	OP287812.1
PP766453	E. dupreanum kobuvirus	*Eidolon dupreanum*	35	97.54	WBP49885.1	*Eidolon dupreanum*	99	99.12	OR082796.1
PP766454	E. dupreanum kobuvirus	*Eidolon dupreanum*	85	99.81	WBP49885.1	*Eidolon dupreanum*	100	98.45	OP287812.1
**PP766456**	E. dupreanum kobuvirus	*Eidolon dupreanum*	88	99.71	WBP49885.1	*Eidolon dupreanum*	99	98.4	OP287812.1
**OQ818317**	E. dupreanum kunsagivirus	*Eidolon helvum*	90	91.61	YP_009345896.1	*Eidolon helvum*	95	81.93	NC_033818.1
**OQ818316**	P. rufus mischivirus	*Hipposideros gigas*	81	46.51	YP_009121743.1	*Suncus murinus*	10	69.71	OQ716013.1
OQ818325	R. madagascariensis picornavirus 1	*Rousettus aegyptiacus*	91	79.35	XBH24017.1	*Rousettus aegyptiacus*	43	73.96	PP711945.1
**OQ818328**	R. madagascariensis picornavirus 1	*Rousettus aegyptiacus*	91	79.7	XBH24017.1	*Rousettus aegyptiacus*	97	73.02	PP711913.1
OQ818346	R. madagascariensis picornavirus 2	*Rousettus aegyptiacus*	99	81.82	XBH23984.1	*Bos taurus*	15	81.94	ON168930.1
**PP766469**	R. madagascariensis picornavirus 3	*Rousettus aegyptiacus*	92	79.03	XBH24017.1	*Rousettus aegyptiacus*	51	72.67	PP711909.1
PP766471	R. madagascariensis picornavirus 3	*Rousettus aegyptiacus*	82	82.11	XBH23984.1	*Rousettus aegyptiacus*	99	74.6	PP711913.1
PP766472	R. madagascariensis picornavirus 3	*Rousettus aegyptiacus*	99	74.54	XBH24017.1	*Rousettus aegyptiacus*	38	75.11	PP711928.1
PP766475	R. madagascariensis picornavirus 3	*Rousettus aegyptiacus*	99	78.65	XBH24017.1	*Rousettus aegyptiacus*	99	73.99	PP711909.1
**PV788825**	R. madagascariensis picornavirus 4	*Rousettus aegyptiacus*	91	79.15	XBH24017.1	*Rousettus aegyptiacus*	90	73.57	PP711909.1
**OQ818320**	E. dupreanum sapelovirus 1	*Eidolon helvum*	96	98.95	YP_009345901.1	*Eidolon helvum*	99	94.37	KX644938.1
OQ818342	E. dupreanum sapelovirus 1	*Eidolon helvum*	99	98.47	YP_009345901.1	*Eidolon helvum*	99	94.73	KX644938.1
OQ818343	E. dupreanum sapelovirus 1	*Eidolon helvum*	99	98.67	YP_009345901.1	*Eidolon helvum*	100	92.28	KX644938.1
OQ818344	E. dupreanum sapelovirus 1	*Eidolon helvum*	99	99.15	YP_009345901.1	*Eidolon helvum*	99	94.57	KX644938.1
PP766465	E. dupreanum sapelovirus 1	*Eidolon helvum*	99	99.19	YP_009345901.1	*Eidolon helvum*	100	94.24	KX644938.1
PP766466	E. dupreanum sapelovirus 1	*Eidolon helvum*	99	98.91	YP_009345901.1	*Eidolon helvum*	100	92.83	KX644938.1
**OQ818321**	E. dupreanum sapelovirus 2	*Eidolon helvum*	83	97.81	XBH23993.1	*Eidolon helvum*	87	87.42	PP711921.1
PP766462	E. dupreanum sapelovirus 2	*Eidolon helvum*	99	97.03	XBH23993.1	*Eidolon helvum*	100	86.55	PP711921.1
PP766463	E. dupreanum sapelovirus 2	*Eidolon helvum*	99	98.31	XBH23993.1	*Eidolon helvum*	100	88.18	PP711921.1
PP766464	E. dupreanum sapelovirus 2	*Eidolon helvum*	99	98.45	XBH23993.1	*Eidolon helvum*	100	88.58	PP711921.1
**OQ818329**	R. madagascariensis sapelovirus 1	*Rousettus aegyptiacus*	94	89.68	XBH23983.1	*Rousettus aegyptiacus*	97	79.04	PP711911.1
**OQ818318**	E. dupreanum teschovirus 1	*Rousettus aegyptiacus*	90	73.14	XBH24020.1	*Rousettus aegyptiacus*	32	72.67	PP711948.1
**OQ818323**	R. madagascariensis teschovirus 1	*Rousettus aegyptiacus*	87	92.16	XBH24006.1	*Rousettus aegyptiacus*	94	82.69	PP711934.1
**OQ818324**	R. madagascariensis teschovirus 2	*Rousettus aegyptiacus*	88	92.25	XBH24006.1	*Rousettus aegyptiacus*	94	79.46	PP711934.1
PV788826	R. madagascariensis teschovirus 2	*Rousettus aegyptiacus*	91	92.16	XBH24006.1	*Rousettus aegyptiacus*	98	79.41	PP711934.1

Supplementary Table 4: Summary table of BLASTx and BLASTn results of novel full and partial-length sapoviruses recovered from mNGS. Bold denotes full-length sequences.

Accession	Virus	BLASTx host species	BLASTx coverage (%)	BLASTx identity (%)	BLASTx accession	BLASTn host species	BLASTn coverage (%)	BLASTn identity (%)	BLASTn accession
OQ818319	E. dupreanum sapovirus 1	*Eidolon helvum*	90	67.18	AQQ78883.1	*Homo sapiens*	1	79.82	LC504397.1
**PP766459**	E. dupreanum sapovirus 1	*Eidolon helvum*	77	99.9	AQQ78883.1	*Homo sapiens*	1	79.82	LC504397.1
OQ818340	E. dupreanum sapovirus 2	*Eidolon helvum*	99	67.97	AQQ78883.1	*Eidolon helvum*	63	71.4	KX759619.1
PP766461	E. dupreanum sapovirus 3	*Eidolon helvum*	96	90.13	AQQ78883.1	*Eidolon helvum*	92	80.7	KX759623.1
PP766460	E. dupreanum sapovirus 4	*Eidolon helvum*	70	75.26	AQQ78883.1	No result	No result	No result	No result
OQ818345	R. madagascariensis sapovirus 1	*Rousettus aegyptiacus*	99	68.19	XBH24168.1	No result	No result	No result	No result
OQ818347	R. madagascariensis sapovirus 2	*Rousettus aegyptiacus*	99	89.63	XBH24156.1	*Rousettus aegypticus*	100	77.94	PP712001.1
PP766470	R. madagascariensis sapovirus 2	*Rousettus aegyptiacus*	99	90.86	XBH24177.1	*Rousettus aegypticus*	99	78.51	PP712015.1
PP766473	R. madagascariensis sapovirus 2	*Rousettus aegyptiacus*	99	90.59	XBH24177.1	*Rousettus aegypticus*	97	77.28	PP712015.1
PP766474	R. madagascariensis sapovirus 2	*Rousettus aegyptiacus*	100	94.55	XBH24177.1	*Rousettus aegypticus*	68	71.74	PP712026.1
PP766476	R. madagascariensis sapovirus 2	*Rousettus aegyptiacus*	99	86.05	XBH24177.1	*Eidolon helvum*	19	73.71	KX759623.1
PP766477	R. madagascariensis sapovirus 2	*Rousettus aegyptiacus*	53	81.32	XBH24178.1	*Rousettus aegypticus*	72	76.22	PP712001.1
OQ818348	R. madagascariensis sapovirus 3	*Rousettus aegyptiacus*	99	84.12	XBH24163.1	*Rousettus aegypticus*	94	75	PP712006.1
PP766468	R. madagascariensis sapovirus 3	*Rousettus aegyptiacus*	100	85	XBH24156.1	*Rousettus aegypticus*	87	75.67	PP712033.1
PV788824	R. madagascariensis sapovirus 4	*Rousettus aegyptiacus*	93	66.89	XBH24168.1	*Rousettus aegyptiacus*	2	79.05	PP712008.1

Supplementary Table 5: Peptide cleavage sites for full and partial-length picornaviruses described in this study. Bold denotes full-length sequences.

Accession	Virus	L/VP4 or VP0	VP4/VP2	VP2 or VP0/VP3	VP3/VP1	VP1/2A	2A/2B	2B/2C	2C/3A	3A/3B	3B/3C	3C/3D
PP766467	E. dupreanum cardiovirus	-	-	-	-	-	-	qqG/Spl	vaQ/Apv	qeQ/Gpy	diQ/Gpn	epQ/Gal
OQ818337	E. dupreanum hepatovirus	-	tlA/Die	mtQ/Mmr	ttQ/Agd	kfE/Eel	ssE/Ase	kaE/Sld	wsQ/Gfs	ltT/Gvy	dsQ/Svv	-
PP766455	E. dupreanum hepatovirus	-	-	-	-	-	-	-	lfQ/Ggv	gsQ/Gpy	-	-
PP766457	E. dupreanum hepatovirus	kpN/Fah	tlK/Spt	saQ/Gfp	-	-	-	-	-	-	-	-
PP766458	E. dupreanum hepatovirus	-	-	-	-	-	-	-	lfQ/Ggv	gsQ/Gpy	enQ/Gpd	giQ/Gvi
PP766449	E. dupreanum kobuvirus	sgQ/Gty	llA/Qps	wrG/Sit	svQ/Dsn	vqQ/Apk	npG/Pai	nkQ/Gkl	skQ/Apt	hnQ/Gpy	elQ/Apg	akQ/Gki
PP766451	E. dupreanum kobuvirus	kpM/Yah	alK/Spt	aaQ/Gip	ffQ/Glg	yyQ/Eqa	veQ/Gll	eeQ/Gpt	lfQ/Gpg	gqQ/Gpy	evQ/Gpd	nfQ/Gsi
PP766452	E. dupreanum kobuvirus	qrQ/Gns	-	akQ/Hwk	ssQ/Ags	vkQ/Gat	rrQ/Gll	qtQ/Glr	krQ/Grv	qpQ/Aay	qrG/Gms	qqQ/Sli
PP766453	E. dupreanum kobuvirus	tqC/Gva	alK/Spt	srQ/Gvp	yfQ/Glg	isQ/Tpi	meQ/Gpm	lpQ/Gvs	lfQ/Gpv	ssQ/Gpy	vtQ/Gpd	leQ/Gei
PP766454	E. dupreanum kobuvirus	rqC/Gva	alK/Spt	mrQ/Gvp	yfQ/Glg	igQ/Tpi	meQ/Gpv	vqQ/Gvs	lfQ/Gpp	snQ/Gpy	vtQ/Gpd	leQ/Gqi
PP766456	E. dupreanum kobuvirus	hkQ/Gvg	llA/Dpk	ytE/Gpa	gtQ/Gpe	vqQ/Gpr	npG/Pai	nkQ/Gkl	skQ/Apq	hnQ/Gpy	elQ/Apg	tkQ/Gki
**OQ818317**	E. dupreanum kunsagivirus	-	-	rrQ/Ifs	lyH/App	aaQ/Gpr	iyG/Pti	ipQ/Gpf	faQ/Sps	psQ/Gpy	dpQ/Gpw	raQ/Gqi
**OQ818316**	P. rufus mischivirus	mfQ/Gag	-	LRQ/Pkl	slQ/Tsl	ttE/Gsp	npG/Pet	klQ/Pll	eiQ/Tke	enQ/Apy	neQ/Kpr	lsE/Gvd
OQ818325	R. madagascariensis picornavirus 1	rqC/Gva	alK/Spt	mrQ/Gvp	yfQ/Glg	igQ/Tpi	meQ/Gpv	vqQ/Gvs	lfQ/Gpp	snQ/Gpy	vtQ/Gpd	leQ/Gqi
**OQ818328**	R. madagascariensis picornavirus 1	rqC/Gva	alK/Spt	mrQ/Gvp	yfQ/Glg	igQ/Tpi	meQ/Gpv	vqQ/Gvs	lfQ/Gpp	snQ/Gpy	vtQ/Gpd	leQ/Gqi
OQ818346	R. madagascariensis picornavirus 2	-	tlA/Die	mtQ/Mmr	ttQ/Agd	kfE/Eel	ssE/Ase	kaE/Sld	wsQ/Gfs	ltT/Gvy	dsQ/Svv	-
**PP766469**	R. madagascariensis picornavirus 3	kpM/Yah	alK/Spt	aaQ/Gip	ffQ/Glg	yyQ/Eqa	veQ/Gll	eeQ/Gpt	lfQ/Gpg	gqQ/Gpy	evQ/Gpd	nfQ/Gsi
PP766471	R. madagascariensis picornavirus 3	-	-	-	-	-	-	-	lfQ/Ggv	gsQ/Gpy	-	-
PP766472	R. madagascariensis picornavirus 3	kpN/Fah	tlK/Spt	saQ/Gfp	-	-	-	-	-	-	-	-
PP766475	R. madagascariensis picornavirus 3	-	-	-	-	-	-	-	lfQ/Ggv	gsQ/Gpy	enQ/Gpd	giQ/Gvi
**PV788825**	R. madagascariensis picornavirus 4	tqC/Gva	alK/Spt	srQ/Gvp	yfQ/Glg	isQ/Tpi	meQ/Gpl	lpQ/Gvs	lfQ/Gpv	ssQ/Gpy	vtQ/Gpd	leQ/Gei
**OQ818320**	E. dupreanum sapelovirus 1	kpN/Fah	tlK/Spt	saQ/Gfp	dlQ/Gfi	irL/Gpp	vyQ/Gik	eeQ/Gft	lfQ/Ggv	gsQ/Gpy	evQ/Gpd	giQ/Gvi
OQ818342	E. dupreanum sapelovirus 1	hkQ/Gvg	llA/Dpk	ytE/Gpa	gtQ/Gpe	vqQ/Gpr	npG/Pai	nkQ/Gkl	skQ/Apq	hnQ/Gpy	elQ/Apg	tkQ/Gki
OQ818343	E. dupreanum sapelovirus 1	sgQ/Gty	llA/Qps	wrG/Sit	svQ/Dsn	vqQ/Apk	npG/Pai	nkQ/Gkl	skQ/Apt	hnQ/Gpy	elQ/Apg	akQ/Gki
OQ818344	E. dupreanum sapelovirus 1	rqC/Gva	alK/Spt	mrQ/Gvp	yfQ/Glg	igQ/Tpi	meQ/Gpv	vqQ/Gvs	lfQ/Gpp	snQ/Gpy	vtQ/Gpd	leQ/Gqi
PP766465	E. dupreanum sapelovirus 1	rqC/Gva	alK/Spt	mrQ/Gvp	yfQ/Glg	igQ/Tpi	meQ/Gpv	vqQ/Gvs	lfQ/Gpp	snQ/Gpy	vtQ/Gpd	leQ/Gqi
PP766466	E. dupreanum sapelovirus 1	-	tlA/Die	mtQ/Mmr	ttQ/Agd	kfE/Eel	ssE/Ase	kaE/Sld	wsQ/Gfs	ltT/Gvy	dsQ/Svv	-
**OQ818321**	E. dupreanum sapelovirus 2	kpN/Iah	alK/Spt	stQ/Gip	evQ/Gfv	irL/Gpp	eiQ/Gik	eeQ/Glv	lfQ/Gpi	gsQ/Gpy	evQ/Gpd	sfQ/Gki
PP766462	E. dupreanum sapelovirus 2	kpM/Yah	alK/Spt	aaQ/Gip	ffQ/Glg	yyQ/Eqa	veQ/Gll	eeQ/Gpt	lfQ/Gpg	gqQ/Gpy	evQ/Gpd	nfQ/Gsi
PP766463	E. dupreanum sapelovirus 2	qrQ/Gns	-	akQ/Hwk	ssQ/Ags	vkQ/Gat	rrQ/Gll	qtQ/Glr	krQ/Grv	qpQ/Aay	qrG/Gms	qqQ/Sli
PP766464	E. dupreanum sapelovirus 2	tqC/Gva	alK/Spt	srQ/Gvp	yfQ/Glg	isQ/Tpi	meQ/Gpm	lpQ/Gvs	lfQ/Gpv	ssQ/Gpy	vtQ/Gpd	leQ/Gei
**OQ818329**	R. madagascariensis sapelovirus 1	qrQ/Gns	-	akQ/Hwk	ssQ/Ags	vkQ/Gat	rrQ/Gll	qtQ/Glr	krQ/Grv	qpQ/Aay	qrG/Gms	qqQ/Sli
**OQ818318**	E. dupreanum teschovirus 1	hkQ/Gag	lsS/Gln	alQ/Gpi	siQ/Gnt	tkQ/Gat	npG/Ppv	kkQ/Gll	tkQ/Apk	keQ/Say	qlQ/Apg	ekQ/Gki
**OQ818323**	R. madagascariensis teschovirus 1	hkQ/Gvg	lsS/Gln	ytE/Gpa	gtQ/Gpe	vqQ/Gpr	npG/Pai	nkQ/Gkl	skQ/Apq	hnQ/Gpy	elQ/Apg	tkQ/Gki
**OQ818324**	R. madagascariensis teschovirus 2	hkQ/Gvg	lsS/Gln	ytE/Gpa	gvQ/Spd	vqQ/Apk	npG/Pai	nkQ/Gkl	skQ/Apq	hnQ/Gpy	elQ/Apg	akQ/Gki
PV788826	R. madagascariensis teschovirus 2	hkQ/Gvg	lsS/Gln	ytE/Gpa	gvQ/Spd	vqQ/Apk	npG/Pai	nkQ/Gkl	skQ/Apq	hnQ/Gpy	elQ/Apg	akQ/Gki

Supplementary Table 6: Conserved motifs in novel picornaviruses from Madagascar fruit bats. Bold denotes full-length sequences. Dashes indicate that the sequence recovered does not include that motif due to length. Absent motifs are otherwise noted.

Accession	Virus species	2C helicase **GxxGxGKS**	2A protease **GxCG**	3C protease **GxCG**	RdRp **KDELR**	RdRp **YGDD**	RdRp **FLKR**
PP766467	E. dupreanum cardiovirus	G_{180}DAGQGKS	-	G_{651}WCG	K_{858}DELR	Y_{1030}GDD	F_{1078}LKR
OQ818337	E. dupreanum hepatovirus	G_{1459}NRGGGKS	-	G_{1924}MCG	-	-	-
PP766455	E. dupreanum hepatovirus	G_{1426}NRGGGKS	-	G_{1891}MCG	K_{2109}DELR	Y_{2285}GDD	F_{2338}LKR
PP766457	E. dupreanum hepatovirus	G_{1459}NRGGGKS	-	G_{1924}MCG	K_{2142}DELR	Y_{2318}GDD	F_{2371}LKR
PP766458	E. dupreanum hepatovirus	-	-	-	K_{216}DELR	Y_{392}GDD	F_{445}LKR
PP766449	E. dupreanum kobuvirus	-	-	-	-	-	-
PP766451	E. dupreanum kobuvirus	G_{908}PPGTGKS	-	-	-	-	-
PP766452	E. dupreanum kobuvirus	-	-	G_{306}LCG	K_{515}DELR	-	-
PP766453	E. dupreanum kobuvirus	-	-	-	-	-	-
PP766454	E. dupreanum kobuvirus	-	-	-	-	-	-
**PP766456**	E. dupreanum kobuvirus	G_{1681}PPGTGKS	-	G_{2145}LCG	K_{2354}DELR	Y_{2522}GDD	F_{2571}LKR
**OQ818317**	E. dupreanum kunsagivirus	G_{1337}EPGTGKS	-	G_{1840}MCG	K_{2053}DELR	Y_{2219}GDD	F_{2267}LKR
**OQ818316**	P. rufus mischivirus	G_{1614}KPGAGKS	-	G_{2085}YCG	K_{2298}DEIR	Y_{2470}GDD	F_{2519}LKR
OQ818325	R. madagascariensis picornavirus 1	G_{1556}SPGCGKS	G_{1272}VCG	G_{2023}QCG	K_{2220}DELR	Y_{2389}GDD	-
**OQ818328**	R. madagascariensis picornavirus 1	G_{1534}SPGCGKS	G_{1250}VCG	G_{2001}QCG	K_{2198}DELR	Y_{2367}GDD	F_{2414}LKR
OQ818346	R. madagascariensis picornavirus 2	G_{36}SPGCGKS	-		-	-	-
**PP766469**	R. madagascariensis picornavirus 3	G_{1492}SPGSGKS	G_{1208}VCG	G_{1960}QCG	K_{2157}DELR	Y_{2326}GDD	F_{2373}LKR
PP766471	R. madagascariensis picornavirus 3	-	-	-	-	-	-
PP766472	R. madagascariensis picornavirus 3	G_{689}QPGSGKS	G_{405}VCG	-	-	-	-
PP766475	R. madagascariensis picornavirus 3	-	-	G_{329}QCG	-	-	-
**PV788825**	R. madagascariensis picornavirus 4	G_{1555}SPGSGKS	G_{1271}VCG	G_{2023}QCG	K_{2220}DELR	Y_{2389}GDD	F_{2436}LKR
**OQ818320**	E. dupreanum sapelovirus 1	G_{1559}SPGTGKS	G_{1243}FCG	G_{2035}QCG	K_{2230}DELR	Y_{2399}GDD	F_{2447}LKR
OQ818342	E. dupreanum sapelovirus 1	G_{67}SPGTGKS	-	-	-	-	-
OQ818343	E. dupreanum sapelovirus 1	-	-	-	-	-	-
OQ818344	E. dupreanum sapelovirus 1	-	-	G_{412}QCG	K_{607}DELR	-	-
PP766465	E. dupreanum sapelovirus 1	G_{320}SPGTGK	G_{4}FCG	-	-	-	-
PP766466	E. dupreanum sapelovirus 1	-	-	-	-	-	-
**OQ818321**	E. dupreanum sapelovirus 2	G_{1577}SPGTGKS	G_{1262}FCG	G_{2051}QCG	K_{2246}DELR	Y_{2415}GDD	F_{2463}LKR
PP766462	E. dupreanum sapelovirus 2	-	G_{510}FCG	-	-	-	-
PP766463	E. dupreanum sapelovirus 2	-	-	-	-	-	-
PP766464	E. dupreanum sapelovirus 2	G_{43}SPGTGK	-	-	-	-	-
**OQ818329**	R. madagascariensis sapelovirus 1	G_{1566}TPGTGKS	G_{1247}YCG	G_{2036}QCG	K_{2231}DELR	Y_{2400}GDD	F_{2448}LKR
**OQ818318**	E. dupreanum teschovirus 1	G_{1368}KPGQGKS	-	G_{1854}YCG, K_{1869}ICG	K_{2063}DELR	Y_{2228}GDD	F_{2275}LKR
**OQ818323**	R. madagascariensis teschovirus 1	G_{1407}KPGQGKS	-	G_{1897}YCG, K_{1912}ICG	K_{2106}DELR	Y_{2272}GDD	F_{2319}LKR
**OQ818324**	R. madagascariensis teschovirus 2	G_{1412}KPGQGKS	-	G_{1902}FCG, K_{1917}ICG	K_{2111}DELR	Y_{2277}GDD	F_{2324}LKR
PV788826	R. madagascariensis teschovirus 2	G_{1410}KPGQGKS	-	G_{1900}FCG, K_{1915}ICG	K_{2109}DELR	Y_{2275}GDD	F_{2322}LKR

Supplementary Table 7: Peptide cleavage sites for full and partial-length sapoviruses described in this study. Bold denotes full-length sequences.

Accession	Virus	NS1 and NS2/Helicase	Helicase/NS4	NS4/Vpg	Vpg/Pro-Pol
OQ818319	E. dupreanum sapovirus 1	-	eaQ/Agk	giE/Akg	esQ/Ags
**PP766459**	E. dupreanum sapovirus 1	qpQ/Aia	eaQ/Agk	giE/Akg	esQ/Ags
OQ818340	E. dupreanum sapovirus 2	-	eaQ/Agk	gvE/Akg	esQ/Ant
PP766461	E. dupreanum sapovirus 3	-	-	-	-
PP766460	E. dupreanum sapovirus 4	-	-	-	-
OQ818345	R. madagascariensis sapovirus 1	-	-	-	-
OQ818347	R. madagascariensis sapovirus 2	-	-	gkK/Gkt	epE/Sgd
PP766470	R. madagascariensis sapovirus 2	-	-	tdE/Akg	epE/San
PP766473	R. madagascariensis sapovirus 2	-	-	tdE/Akg	epE/San
PP766474	R. madagascariensis sapovirus 2	-	eaQ/Apn	-	-
PP766476	R. madagascariensis sapovirus 2	-	-	-	-
PP766477	R. madagascariensis sapovirus 2	-	-	-	-
OQ818348	R. madagascariensis sapovirus 3	-	fpQ/Ssd	eeE/Akg	idE/Gps
PP766468	R. madagascariensis sapovirus 3	-	eaQ/Sgn	-	-
PV788824	R. madagascariensis sapovirus 4	tdA/Gla	ppQ/Rqi	vlE/Gkk	ieE/Gsa

Supplementary Table 8: Conserved motifs in novel Sapovirus sequences from Madagascar fruit bats. Bold denotes full-length sequences. Dashes indicate that the sequence recovered does not include that motif due to length. Absent motifs are otherwise noted.

**Accession**	**Virus species**	NTPase **GAPGIGKT**	Vpg **KGKTK/ DDEYDE**	Protease **GxCG**	RdRp **WKGL/ KDELR**	RdRp **DYSKWDST**	RdRp **GLPSG/ YGDD**	Vp1 **PPG/ GWS**
OQ818319	E. dupreanum sapovirus 1	G_{140}PPGIGKT	K_{609}GKTK/ D_{624}DEYEE	G_{832}DCG	W_{877}KGL/ K_{1039}DELR	D_{1114}YSKWDST	G_{1169}LPSG/ Y_{1217}GDD	P_{1510}PG/G_{1656}WS
**PP766459**	E. dupreanum sapovirus 1	G_{469}PPGIGKT	K_{938}GKTK/ D_{953}DEYEE	G_{1161}DCG	W_{1206}KGL/ K_{1368}DELR	D_{1443}YSKWDST	G_{1498}LPSG/ Y_{1546}GDD	P_{1839}PG/G_{1985}WS
OQ818340	E. dupreanum sapovirus 2	G_{201}PPGIGKT	K_{670}GKTK/ D_{685}DEYEE	G_{893}DCG	W_{938}KGL	-	-	-
PP766461	E. dupreanum sapovirus 3	-	-	-	-	-	G_{39}LPSG/ Y_{87}GDD	P_{379}PG/ G_{525}WS
PP766460	E. dupreanum sapovirus 4	-	-	-	-	-	-	
OQ818345	R. madagascariensis sapovirus 1	-	-	-	-	-	-	P_{138}PG/ G_{283}WS
OQ818347	R. madagascariensis sapovirus 2	-	K_{89}GKTK/ D_{106}DEYEE	G_{314}DCG	W_{359}KGL/ K_{521}DELR	D_{596}FSKWDST	G_{651}LPSG/ Y_{699}GDD	-
PP766470	R. madagascariensis sapovirus 2	-	K_{121}GKTK/ D_{137}DEYEE	G_{345}DCG	W_{390}KGL/ K_{552}DELR	D_{627}YSKWDST	G_{682}LPSG/ Y_{730}GDD	-
PP766473	R. madagascariensis sapovirus 2	-	K_{16G}KTK/ D_{32}DEYEE	G_{240}DCG	W_{285}KGL/ K_{447}DELR	D_{522}YSKWDST	G_{577}LPSG/ Y_{625}GDD	-
PP766474	R. madagascariensis sapovirus 2	G_{103}PPGIGKT	-	-	-	-	-	-
PP766476	R. madagascariensis sapovirus 2	-	-	-	-	-	-	P_{42}PG/ G_{188}WS
PP766477	R. madagascariensis sapovirus 2	-	-	-	-	-	-	-
OQ818348	R. madagascariensis sapovirus 3	-	Absent/ D_{287}DEYEE	G_{472}SCG	W_{519}KGL/ K_{679}DELR	D_{754}YSKWDST	G_{809}LPSG/ Y_{857}GDD	-
PP766468	R. madagascariensis sapovirus 3	G_{28}PPGIGKT	-	-	-	-	-	-
PV788824	R. madagascariensis sapovirus 4	G_{473}PPGVGKS	K_{946}KKGK/D_{960}DEYEE	G_{1146}DCG	W_{1193}KGL/K_{1354}DELR	D_{1429}YSKWDST	G_{1484}LPSG/ Y_{1532}GDD	P_{1829}PG/G_{1974}WS

Supplementary Table 9: : RDP4 output of comparisons involving novel sequences as either the recombinant sequence or a major/minor parental sequence. Unknown followed by an accession number indicates that RDP4 used that named sequence to infer an unknown parental sequence. NS denotes non-significance in that analysis. Recombination events are accepted if at least 4 tests are significant. Recombinant sequences with highest support are highlighted in red (5-6 significant tests). Novel sequences identified in this study are in bold. Particular lineages are consensus sequences of aligned phylogenetic clade members and include: Rhinolophus bat picornavirus BtSY4 clade (accessions OP963617 and PP746000), *Shanbavirus* A clade (accessions KJ641687, KJ641690, KJ641699, and KJ641693), *R. aegypticus* African picornavirus clade (accessions PP711912, PP711945, PP711913, PP711930, PP711928, and PP711909), *H. larvatus* Chinese *Hepatovirus* clade (accessions OR951274, OR951275, OR867086, OR951271), *Rattus tanezumi* Chinese *Kobuvirus* clade (accessions OM069746 and OM069755), *Rhinolophus* Chinese *Kobuvirus* clade (accessions OR951245 and OR951365), Rodent Chinese *Kunsagivirus* clade (accessions OQ716009 and OQ716008), *Eonycteris* Chinese *Sapelovirus* clade (accessions OR951327, OR951326, OR951325, and OR951332), *E. helvum* Cameroonian *Sapovirus* clade (accessions KX759619, KX759623, KX759621, NC_033776, and KX759622), *R. aegypticus* Kenyan *Sapovirus* clade (accessions PP712001, PP712004, PP712008, and PP712015), *R. aegypticus* east African *Teschovirus* clade (accessions PP711948 and PP711934), and *Rousettus* Chinese *Teschovirus* clade (accessions OR951335, OR951333, and OR951334).

Alignment file	Genus	Breakpoint Begin	Breakpoint End	Recombinant Sequence	Major Parental Sequence	Minor Parental Sequence	RDP	GENECONV	Bootscan	Maxchi	Chimaera	3Seq	Number significant tests	Accept Recombination Event?	Corresponding Figure
batpicorna_rdp_align_nt_clade	*Bat picornavirus*	117	184	rhinolophus_BtSY4	**Unknown (OQ818328)**	PP711912	7.56E-03	NS	NS	8.86E-03	1.28E-03	NS	2	no	
batpicorna_rdp_align_nt_clade	*Bat picornavirus*				**Unknown(OQ818325)**										
batpicorna_rdp_align_nt_clade	*Bat picornavirus*	6885	7153	rhinolophus_BtSY4	**OQ818325**	**Unknown (OQ818328)**	NS	NS	9.66E-03	NS	NS	NS	1	no	
batpicorna_rdp_align_nt_clade	*Bat picornavirus*	3238	3452	**PP766469**	PP711912	**Unknown (OQ818325)**	1.70E-02	NS	2.07E-02	NS	NS	NS	2	no	
batpicorna_rdp_align_nt_clade	*Bat picornavirus*	962	1311	**PP766469**	rhinolophus_BtSY4	**OQ818325**	NS	NS	1.27E-02	3.42E-04	1.71E-02	NS	3	no	
batpicorna_rdp_align_nt_clade	*Bat picornavirus*					**OQ818328**									
batpicorna_rdp_align_nt_clade	*Bat picornavirus*	1548	1709	rhinolophus_BtSY4	shanbavirus_a	**PP766469**	1.52E-02	NS	NS	NS	NS	NS	1	no	
batpicorna_rdp_align_nt	*Bat picornavirus*	7547	104	**PV788825**	Unknown (PP711909)	PP711912	3.23E-05	NS	NS	0.000195944	0.00034979	0.03732805	4	yes	Supplementary Fig. 7A
batpicorna_rdp_align_nt	*Bat picornavirus*				Unknown(PP711930)	PP711913									
batpicorna_rdp_align_nt	*Bat picornavirus*	814	1160	**PV788825**	PP711913	Unknown (PP711930)	7.91E-03	NS	0.021952624	0.010595024	0.000387329	0.043266161	5	yes	
batpicorna_rdp_align_nt	*Bat picornavirus*					Unknown(PP711909)									
batpicorna_rdp_align_nt	*Bat picornavirus*	844	1148	OQ363775.1	Unknown (PP746000)	**PP766469**	2.21E-04	NS	NS	NS	3.30E-03	NS	2	no	
batpicorna_rdp_align_nt	*Bat picornavirus*			HQ595345.1											
batpicorna_rdp_align_nt	*Bat picornavirus*			NC_015934.1											
batpicorna_rdp_align_nt	*Bat picornavirus*			KJ641693.1											
batpicorna_rdp_align_nt	*Bat picornavirus*			OQ363764.1											
batpicorna_rdp_align_nt	*Bat picornavirus*	1365	1464	PP711930	**OQ818328**	KJ641687.1	6.84E-04	NS	NS	2.84E-03	1.74E-04	NS	3	no	
batpicorna_rdp_align_nt	*Bat picornavirus*			PP711909	**OQ818325**	KJ641690.1									
batpicorna_rdp_align_nt	*Bat picornavirus*	3218	4049	HQ595345.1	**OQ818328**	**Unknown (PP766469)**	6.01E-04	3.60E-03	NS	7.20E-04	4.10E-02	NS	4	yes	
batpicorna_rdp_align_nt	*Bat picornavirus*			NC_015934.1											
batpicorna_rdp_align_nt	*Bat picornavirus*			OQ363775.1											
batpicorna_rdp_align_nt	*Bat picornavirus*	5057	5220	**PP766469**	**OQ818328**	Unknown (PP746000)	7.56E-04	NS	NS	NS	NS	NS	1	no	
batpicorna_rdp_align_nt	*Bat picornavirus*	1588	1689	PP746000	OP963617.1	**PP766469**	1.97E-03	NS	NS	NS	NS	NS	1	no	
batpicorna_rdp_align_nt	*Bat picornavirus*	330	554	**OQ818328**	**PP766469**	OP963617.1	3.55E-03	NS	NS	NS	NS	0.016164469	2	no	
batpicorna_rdp_align_nt	*Bat picornavirus*			**OQ818325**											
batpicorna_rdp_align_nt	*Bat picornavirus*	4688	4860	**OQ818328**	**PP766469**	PP745912	6.88E-03	NS	NS	NS	NS	4.62E-02	2	no	
batpicorna_rdp_align_nt	*Bat picornavirus*			**OQ818325**											
batpicorna_rdp_align_nt	*Bat picornavirus*	6704	6859	PP745912	Unknown (HQ595345.1)	**OQ818328**	5.38E-03	NS	3.43E-02	NS	NS	NS	2	no	
batpicorna_rdp_align_nt	*Bat picornavirus*				Unknown(NC_015934.1)										
batpicorna_rdp_align_nt	*Bat picornavirus*	7387	34	PP711912	PP711930	**Unknown (OQ818328)**	1.35E-02	NS	0.018845876	1.01E-02	5.34E-03	NS	4	yes	
batpicorna_rdp_align_nt	*Bat picornavirus*	117	201	OP963617.1	**OQ818328**	Unknown (PP711945)	0.019271697	NS	NS	NS	NS	NS	1	no	
batpicorna_rdp_align_nt	*Bat picornavirus*	3262	3281	**OQ818325**	PP711928	Unknown (OQ363764.1)	2.23E-02	NS	NS	NS	NS	NS	1	no	
batpicorna_rdp_align_nt	*Bat picornavirus*			**OQ818328**											
batpicorna_rdp_align_nt	*Bat picornavirus*	5268	5303	**OQ818328**	PP711928	HQ595345.1	2.36E-02	NS	NS	NS	NS	NS	1	no	
batpicorna_rdp_align_nt	*Bat picornavirus*			**OQ818325**		NC_015934.1									
batpicorna_rdp_align_nt	*Bat picornavirus*	4854	4882	PP711928	**OQ818328**	KJ641693.1	2.24E-02	NS	NS	NS	NS	NS	1	no	
batpicorna_rdp_align_nt	*Bat picornavirus*				**OQ818325**										
batpicorna_rdp_align_nt	*Bat picornavirus*	6448	6524	OQ363764.1	**PP766469**	KJ641690.1	2.68E-02	NS	NS	NS	NS	NS	1	no	
batpicorna_rdp_align_nt	*Bat picornavirus*			HQ595345.1		KJ641687.1									
batpicorna_rdp_align_nt	*Bat picornavirus*			NC_015934.1		KJ641699.1									
batpicorna_rdp_align_nt	*Bat picornavirus*			OQ363775.1											
batpicorna_rdp_align_nt	*Bat picornavirus*	3859	3987	PP746000	**Unknown (OQ818328)**	KJ641690.1	2.79E-02	NS	NS	NS	NS	NS	1	no	
batpicorna_rdp_align_nt	*Bat picornavirus*	2654	3026	PP711946	Unknown (KJ641690.1)	**PP766469**	4.87E-02	NS	NS	3.90E-02	NS	NS	2	no	
hepato_rdp_align_nt_clade	*Hepatovirus*	7198	560	NC_028366	Unknown (NC_028981)	**PP766455**	1.83E-04	1.46E-06	1.58E-08	6.59E-06	1.51E-02	NS	5	yes	Supplementary Fig. 7B
hepato_rdp_align_nt_clade	*Hepatovirus*					**PP766457**									
hepato_rdp_align_nt_clade	*Hepatovirus*	577	7197	NC_028366	**PP766457**	H_larvatus_china	NS	NS	5.76E-04	6.73E-03	7.99E-03	NS	3	no	
hepato_rdp_align_nt_clade	*Hepatovirus*				**PP766455**										
hepato_rdp_align_nt_clade	*Hepatovirus*	1745	1968	**PP766457**	NC_028366	KT452729	6.03E-03	NS	NS	NS	4.27E-02	4.02E-02	3	no	
hepato_rdp_align_nt_clade	*Hepatovirus*			**PP766455**											
hepato_rdp_align_nt_clade	*Hepatovirus*	1012	1156	NC_038313	Unknown (H_larvatus_china)	**PP766455**	1.52E-02	2.22E-02	NS	9.13E-03	NS	NS	3	no	
hepato_rdp_align_nt_clade	*Hepatovirus*					**PP766457**									
hepato_rdp_align_nt	*Hepatovirus*	7224	560	NC_028366	Unknown (NC_028981)	**PP766455**	1.50E-03	3.63E-05	1.92E-07	1.96E-04	1.36E-04	NS	5	yes	
hepato_rdp_align_nt	*Hepatovirus*					**PP766457**									
hepato_rdp_align_nt	*Hepatovirus*	6939	640	OR867086	**Unknown (PP766455)**	OR951271	NS	5.02E-03	7.66E-05	8.91E-05	NS	NS	3	no	
hepato_rdp_align_nt	*Hepatovirus*			OR951275											
hepato_rdp_align_nt	*Hepatovirus*	3326	4084	NC_038313	OR951275	**Unknown (PP766457)**	5.41E-03	NS	NS	2.66E-02	NS	NS	2	no	
hepato_rdp_align_nt	*Hepatovirus*					**Unknown(PP766455)**									
hepato_rdp_align_nt	*Hepatovirus*	1583	1920	NC_028366	**PP766455**	Unknown (KT452729)	1.09E-02	NS	NS	NS	NS	NS	1	no	
hepato_rdp_align_nt	*Hepatovirus*				**PP766457**										
kobu_rdp_align_nt_clade	*Kobuvirus*	7921	8347	**OQ818322**	OP287812.1	Unknown (NC_034971.1)	NS	NS	3.08E-04	NS	NS	NS	1	no	
kobu_rdp_align_nt_clade	*Kobuvirus*				NC_034971.1	Unknown(OP287812.1)									
kobu_rdp_align_nt_clade	*Kobuvirus*	1482	1817	NC_034971.1	rattus_tanezumi_china	**Unknown (PP766456)**	NS	NS	8.99E-04	NS	NS	4.45E-03	2	no	
kobu_rdp_align_nt_clade	*Kobuvirus*				MN116647	Unknown(OP287812.1)									
kobu_rdp_align_nt_clade	*Kobuvirus*					**Unknown(OQ818322)**									
kobu_rdp_align_nt_clade	*Kobuvirus*	3155	3399	NC_034971.1	rattus_tanezumi_china	**Unknown (PP766456)**	6.57E-04	NS	NS	NS	NS	1.12E-02	2	no	
kobu_rdp_align_nt_clade	*Kobuvirus*					Unknown(OP287812.1)									
kobu_rdp_align_nt_clade	*Kobuvirus*					**Unknown(OQ818322)**									
kobu_rdp_align_nt_clade	*Kobuvirus*	4286	6667	rattus_tanezumi_china	rhinolophus_china	**PP766456**	3.28E-03	NS	NS	NS	NS	NS	1	no	
kobu_rdp_align_nt_clade	*Kobuvirus*			MN116647											
kobu_rdp_align_nt_clade	*Kobuvirus*			NC_034971.1											
kobu_rdp_align_nt_clade	*Kobuvirus*				Unknown(MN116647)	OP287812.1									
kobu_rdp_align_nt_clade	*Kobuvirus*				Unknown(rattus_tanezumi_china)	**PP766456**									
kobu_rdp_align_nt_clade	*Kobuvirus*	2686	2864	NC_034971.1	rattus_tanezumi_china	**OQ818322**	2.10E-02	NS	NS	NS	NS	NS	2	no	
kobu_rdp_align_nt	*Kobuvirus*	3107	3399	NC_034971.1	OR951245.1	**Unknown (PP766456)**	1.56E-04	NS	5.25E-03	NS	NS	3.54E-02	3	no	
kobu_rdp_align_nt	*Kobuvirus*					Unknown(OP287812.1)									
kobu_rdp_align_nt	*Kobuvirus*					**Unknown(OQ818322)**									
kobu_rdp_align_nt	*Kobuvirus*	1482	1817	NC_034971.1	OM069746	**Unknown (OQ818322)**	4.98E-04	NS	5.14E-03	NS	NS	8.45E-03	3	no	
kobu_rdp_align_nt	*Kobuvirus*				MN116647	Unknown(OP287812.1)									
kobu_rdp_align_nt	*Kobuvirus*				OM069755	**Unknown(PP766456)**									
kobu_rdp_align_nt	*Kobuvirus*	7809	8216	NC_034971.1	**OQ818322**	Unknown (OP287812.1)	NS	NS	7.69E-04	NS	NS	NS	1	no	
kobu_rdp_align_nt	*Kobuvirus*	3894	4511	OR951365.1	**Unknown (PP766456)**	NC_034971.1	NS	NS	5.71E-03	5.00E-03	0.003761435	NS	3	no	
kobu_rdp_align_nt	*Kobuvirus*				Unknown(OP287812.1)										
kobu_rdp_align_nt	*Kobuvirus*				**Unknown(OQ818322)**										
kobu_rdp_align_nt	*Kobuvirus*	3872	5081	OR951245.1	**Unknown (OQ818322)**	MN116647	7.54E-03	NS	NS	1.65E-02	8.93E-04	NS	3	no	
kobu_rdp_align_nt	*Kobuvirus*			OR951365.1	**Unknown(PP766456)**										
kobu_rdp_align_nt	*Kobuvirus*	7147	7253	MN116647	OR951245.1	OP287812.1	3.51E-02	2.43E-03	1.02E-02	NS	NS	NS	3	no	
kobu_rdp_align_nt	*Kobuvirus*					**OQ818322**									
kobu_rdp_align_nt	*Kobuvirus*	6619	6724	NC_034971.1	OM069746	**PP766456**	0.011078326	NS	NS	7.54E-03	9.86E-03	NS	3	no	
kobu_rdp_align_nt	*Kobuvirus*	2686	2864	NC_034971.1	OM069755	**OQ818322**	2.26E-02	NS	3.23E-02	NS	NS	NS	2	no	
kobu_rdp_align_nt	*Kobuvirus*	7905	8156	OP287812.1	OR951245.1	Unknown (MN116647)	NS	1.57E-03	4.74E-02	0.02374371	NS	NS	3	no	
kobu_rdp_align_nt	*Kobuvirus*			**OQ818322**	MN116647	Unknown(OR951245.1)									
kobu_rdp_align_nt	*Kobuvirus*			**PP766456**											
kobu_rdp_align_nt	*Kobuvirus*	5085	5593	OR951245.1	**Unknown (PP766456)**	OM069746	3.60E-02	1.52E-02	NS	NS	2.83E-02	NS	3	no	
kunsagi_rdp_align_nt_clade	*Kunsagivirus*					**OQ818317**									
kunsagi_rdp_align_nt_clade	*Kunsagivirus*	1815	1865	NC_033818.1	OP589993.1	NC_034206.1	1.54E-03	NS	NS	NS	NS	NS	1	no	
kunsagi_rdp_align_nt_clade	*Kunsagivirus*			**OQ818317**											
kunsagi_rdp_align_nt_clade	*Kunsagivirus*	4320	4779	NC_034206.1	**OQ818317**	Unknown (rodent_china)	5.16E-03	NS	NS	NS	NS	NS	1	no	
kunsagi_rdp_align_nt	*Kunsagivirus*	5893	5949	NC_038317	OQ716009	**Unknown (OQ818317)**	1.83E-04	1.90E-02	NS	NS	NS	5.50E-03	3	no	
kunsagi_rdp_align_nt	*Kunsagivirus*					Unknown(NC_033818.1)									
kunsagi_rdp_align_nt	*Kunsagivirus*	1814	1865	NC_033818.1	OP589993.1	NC_034206.1	2.92E-03	NS	NS	NS	NS	NS	1	no	
kunsagi_rdp_align_nt	*Kunsagivirus*			**OQ818317**											
kunsagi_rdp_align_nt	*Kunsagivirus*	4893	5027	NC_038317	OQ716008	**Unknown (OQ818317)**	8.00E-03	NS	NS	NS	NS	1.99E-02	2	no	
kunsagi_rdp_align_nt	*Kunsagivirus*	4351	4670	NC_034206.1	NC_038317	**Unknown (OQ818317)**	NS	NS	8.82E-03	7.05E-03	7.58E-03	NS	3	no	
kunsagi_rdp_align_nt	*Kunsagivirus*	3461	3734	NC_033818.1	Unknown (NC_034206.1)	NC_038317	5.24E-03	2.60E-02	3.30E-02	NS	3.44E-02	NS	4	yes	
kunsagi_rdp_align_nt	*Kunsagivirus*			**OQ818317**											
kunsagi_rdp_align_nt	*Kunsagivirus*	5966	6057	**OQ818317**	OQ716009	Unknown (NC_038317)	1.02E-02	NS	NS	NS	NS	NS	1	no	
kunsagi_rdp_align_nt	*Kunsagivirus*				OQ716008										
mischi_rdp_align_nt	*Mischivirus*	5269	5420	NC_026470.1	**OQ818316**	MG888045.1	6.34E-04	NS	3.65E-03	8.00E-03	NS	NS	3	no	
mischi_rdp_align_nt	*Mischivirus*	2021	2127	NC_075428.1	MG888045.1	**Unknown (OQ818316)**	1.44E-02	NS	NS	NS	NS	NS	1	no	
mischi_rdp_align_nt	*Mischivirus*	6142	6198	OR951360.1	NC_043072.1	**OQ818316**	3.86E-02	NS	NS	NS	NS	NS	1	no	
mischi_rdp_align_nt	*Mischivirus*	2128	2388	NC_075428.1	**Unknown (OQ818316)**	NC_043072.1	4.12E-02	NS	NS	NS	NS	NS	1	no	
sapelo_rdp_align_nt_clade	*Sapelovirus*	7443	269	PP711943	Unknown (NC_003987)	**OQ818320**	NS	NS	1.10E-12	4.02E-02	2.41E-06	NS	3	no	
sapelo_rdp_align_nt_clade	*Sapelovirus*	268	817	**OQ818321**	Unknown (PP711911)	PP711943	1.99E-09	1.11E-06	7.83E-07	2.57E-07	2.46E-05	NS	5	yes	Supplementary Fig. 7C
sapelo_rdp_align_nt_clade	*Sapelovirus*					NC_033820									
sapelo_rdp_align_nt_clade	*Sapelovirus*					**OQ818320**									
sapelo_rdp_align_nt_clade	*Sapelovirus*	3071	5027	NC_033820	**OQ818320**	PP711943	6.34E-09	NS	6.77E-06	4.66E-10	2.87E-11	1.87E-14	5	yes	Fig. 5A
sapelo_rdp_align_nt_clade	*Sapelovirus*	101	758	PP711921	Unknown (PP711911)	NC_033820	5.94E-10	8.31E-08	2.98E-07	4.71E-07	1.17E-07	NS	4	yes	
sapelo_rdp_align_nt_clade	*Sapelovirus*					**OQ818320**									
sapelo_rdp_align_nt_clade	*Sapelovirus*	2991	3919	**OQ818329**	PP711921	Unknown (PP711943)	7.65E-08	NS	NS	1.39E-02	6.74E-07	NS	3	no	
sapelo_rdp_align_nt_clade	*Sapelovirus*				**OQ818320**	**Unknown(OQ818321)**									
sapelo_rdp_align_nt_clade	*Sapelovirus*				PP711943	Unknown(PP711921)									
sapelo_rdp_align_nt_clade	*Sapelovirus*			**OQ818329**	**OQ818321**										
sapelo_rdp_align_nt_clade	*Sapelovirus*	2236	2304	PP711911	Unknown (PP711921)	**OQ818320**	1.34E-04	1.34E-03	NS	2.27E-03	2.30E-02	NS	4	yes	
sapelo_rdp_align_nt_clade	*Sapelovirus*					NC_033820									
sapelo_rdp_align_nt_clade	*Sapelovirus*	6799	6863	PP711921	**OQ818321**	**Unknown (OQ818320)**	2.91E-04	6.80E-03	NS	NS	NS	NS	2	no	
sapelo_rdp_align_nt_clade	*Sapelovirus*	6476	6624	**OQ818329**	PP711921	Unknown (NC_003987)	9.19E-04	NS	3.69E-02	NS	NS	NS	2	no	
sapelo_rdp_align_nt_clade	*Sapelovirus*			PP711943											
sapelo_rdp_align_nt_clade	*Sapelovirus*	5119	6539	PP711943	**Unknown (OQ818329)**	NC_033820	NS	1.29E-14	5.87E-14	5.40E-04	4.16E-08	NS	4	yes	
sapelo_rdp_align_nt_clade	*Sapelovirus*					**OQ818320**									
sapelo_rdp_align_nt_clade	*Sapelovirus*	3977	5118	PP711943	**Unknown (OQ818329)**	**OQ818320**	NS	1.14E-15	8.48E-14	4.69E-18	NS	NS	3	no	
sapelo_rdp_align_nt_clade	*Sapelovirus*			**OQ818320**											
sapelo_rdp_align_nt_clade	*Sapelovirus*	6711	7438	PP711943	**Unknown (OQ818329)**	**OQ818320**	NS	8.47E-10	2.67E-07	3.01E-03	1.94E-04	NS	4	yes	
sapelo_rdp_align_nt_clade	*Sapelovirus*	48	441	NC_003987	**OQ818320**	Unknown (PP711911)	7.12E-03	NS	2.90E-03	6.90E-03	NS	NS	3	no	

sapelo_rdp_align_nt_clade	*Sapelovirus*				PP711911	**Unknown(OQ818320)**									
sapelo_rdp_align_nt_clade	*Sapelovirus*	2169	2281	**OQ818329**	**Unknown (OQ818321)**	**OQ818320**	1.25E-02	NS	NS	NS	NS	NS	1	no	
sapelo_rdp_align_nt_clade	*Sapelovirus*					NC_033820									
sapelo_rdp_align_nt_clade	*Sapelovirus*	5647	5711	PP711911	eonycteris_china	**OQ818320**	1.54E-02	NS	NS	NS	NS	NS	1	no	
sapelo_rdp_align_nt_clade	*Sapelovirus*	1273	1313	PP711943	**OQ818321**	NC_003987	1.78E-02	NS	NS	NS	NS	NS	1	no	
sapelo_rdp_align_nt_clade	*Sapelovirus*				PP711921										
sapelo_rdp_align_nt_clade	*Sapelovirus*	2408	2445	**OQ818329**	PP711911	NC_033820	3.73E-02	NS	NS	NS	NS	NS	1	no	
sapelo_rdp_align_nt_clade	*Sapelovirus*	6199	6458	**OQ818321**	NC_033820	PP711911	0.044047578	NS	NS	1.41E-02	NS	NS	2	no	
sapelo_rdp_align_nt_clade	*Sapelovirus*			PP711921											
sapelo_rdp_align_nt_clade	*Sapelovirus*			**OQ818321**											
sapelo_rdp_align_nt	*Sapelovirus*	7443	269	PP711943	Unknown (NC_003987)	**OQ818320**	NS	NS	4.52E-12	6.59E-09	2.04E-05	NS	3	no	
sapelo_rdp_align_nt	*Sapelovirus*	7733	847	**OQ818321**	Unknown (OR951327)	NC_033820	5.57E-08	NS	4.72E-09	7.25E-04	6.45E-08	NS	4	yes	
sapelo_rdp_align_nt	*Sapelovirus*				Unknown(OR951326)										
sapelo_rdp_align_nt	*Sapelovirus*	3070	5027	NC_033820	**OQ818320**	PP711943	1.66E-08	NS	1.77E-05	1.22E-09	7.52E-11	4.88E-14	5	yes	
sapelo_rdp_align_nt	*Sapelovirus*				Unknown(OR951327)	**OQ818320**									
sapelo_rdp_align_nt	*Sapelovirus*			**OQ818321**	Unknown(OR951327)	**OQ818320**									
sapelo_rdp_align_nt	*Sapelovirus*	6796	6862	PP711921	**OQ818321**	**Unknown (OQ818320)**	1.66E-04	9.61E-04	NS	NS	NS	NS	2	no	
sapelo_rdp_align_nt	*Sapelovirus*					Unknown(NC_033820)									
sapelo_rdp_align_nt	*Sapelovirus*	2237	2303	PP711911	Unknown (PP711921)	**OQ818320**	2.20E-04	2.46E-03	NS	4.03E-03	2.50E-02	NS	4	yes	
sapelo_rdp_align_nt	*Sapelovirus*	4437	5660	PP711921	Unknown (OR951326)	**OQ818320**	1.33E-03	NS	NS	5.37E-03	2.71E-03	NS	3	no	
sapelo_rdp_align_nt	*Sapelovirus*			**OQ818321**		PP711943									
sapelo_rdp_align_nt	*Sapelovirus*	6279	6429	NC_003987	**Unknown (OQ818329)**	PP711921	2.32E-03	NS	NS	NS	NS	NS	1	no	
sapelo_rdp_align_nt	*Sapelovirus*	0	384	**OQ818329**	Unknown (NC_003987)	**OQ818320**	3.39E-03	1.78E-02	1.36E-02	1.38E-04	NS	NS	4	yes	
sapelo_rdp_align_nt	*Sapelovirus*			PP711911		NC_033820									
sapelo_rdp_align_nt	*Sapelovirus*	7170	7256	PP711911	OR951326	**OQ818320**	NS	NS	6.53E-03	NS	NS	NS	1	no	
sapelo_rdp_align_nt	*Sapelovirus*					NC_033820									
sapelo_rdp_align_nt	*Sapelovirus*	3143	4199	**OQ818329**	OR951325	Unknown (PP711943)	0.009677487	NS	NS	NS	NS	NS	1	no	
sapelo_rdp_align_nt	*Sapelovirus*				PP711943	Unknown(OR951325)									
sapelo_rdp_align_nt	*Sapelovirus*	1773	1796	PP711943	**OQ818320**	Unknown (OR951332)	1.40E-02	NS	NS	NS	NS	NS	1	no	
sapelo_rdp_align_nt	*Sapelovirus*	1752	1933	OR951325	OR951332	**OQ818320**	1.44E-02	NS	8.25E-03	5.35E-03	9.49E-03	NS	4	yes	
sapelo_rdp_align_nt	*Sapelovirus*	4182	4459	NC_003987	OR951325	Unknown (PP711943)	0.01191445	NS	NS	1.98E-02	1.83E-02	NS	3	no	
sapelo_rdp_align_nt	*Sapelovirus*				**OQ818320**	Unknown(OR951325)									
sapelo_rdp_align_nt	*Sapelovirus*				PP711943										
sapelo_rdp_align_nt	*Sapelovirus*	845	1051	OR951325	Unknown (PP711921)	PP711943	1.79E-02	NS	NS	NS	NS	NS	1	no	
sapelo_rdp_align_nt	*Sapelovirus*	621	639	**OQ818329**	PP711911	PP711943	3.97E-02	NS	NS	NS	NS	NS	1	no	
sapelo_rdp_align_nt	*Sapelovirus*	6465	6500	**OQ818329**	PP711911	NC_033820	4.30E-02	NS	NS	NS	NS	NS	1	no	
sapelo_rdp_align_nt	*Sapelovirus*	2162	2547	**OQ818329**	Unknown (PP711921)	PP711943	NS	NS	2.25E-02	1.42E-02	NS	NS	2	no	
sapo_rdp_align_nt_clade	*Sapovirus*	1140	1451	e_helvum_cameroon	ON872527	OQ709197	5.70E-03	NS	7.73E-03	2.58E-02	NS	NS	3	no	
sapo_rdp_align_nt_clade	*Sapovirus*			**OQ818319**											
sapo_rdp_align_nt_clade	*Sapovirus*			**PP766459**											
sapo_rdp_align_nt_clade	*Sapovirus*			OP963623											
sapo_rdp_align_nt_clade	*Sapovirus*	3225	7029	e_helvum_cameroon	Unknown (r_aegypticus_kenya)	**PP766459**	4.64E-03	NS	NS	3.18E-05	3.97E-05	3.66E-04	4	yes	
sapo_rdp_align_nt_clade	*Sapovirus*	7749	893	r_aegypticus_kenya	OQ709197	**PP766459**	1.29E-02	NS	1.21E-02	1.97E-02	2.03E-04	NS	4	yes	
sapo_rdp_align_nt_clade	*Sapovirus*	196	405	ON872527	Unknown (OQ709197)	**PP766459**	3.91E-02	NS	NS	NS	NS	NS	1	no	
sapo_rdp_align_nt_clade	*Sapovirus*	1983	2066	ON872527	Unknown (OP963622)	**PP766459**	2.44E-02	0.024391296	NS	NS	NS	NS	2	no	
sapo_rdp_align_nt_clade	*Sapovirus*					**OQ818319**									
sapo_rdp_align_nt_clade	*Sapovirus*	6645	6815	OP963623	**PP766459**	OQ709197	4.22E-02	NS	NS	1.68E-03	1.20E-03	3.78E-02	4	yes	
sapo_rdp_align_nt_clade	*Sapovirus*				**OQ818319**										
sapo_rdp_align_nt_clade	*Sapovirus*	5752	6021	OP963623	OP963622	ON872527	4.51E-02	NS	NS	NS	2.81E-02	NS	2	no	
sapo_rdp_align_nt_clade	*Sapovirus*			**OQ818319**											
sapo_rdp_align_nt_clade	*Sapovirus*			**PP766459**											
sapo_rdp_align_nt	*Sapovirus*	4883	5167	KX759623	**PP766459**	PP712001	NS	NS	8.36E-04	NS	NS	7.22E-03	2	no	
sapo_rdp_align_nt	*Sapovirus*				**OQ818319**										
sapo_rdp_align_nt	*Sapovirus*	2870	2948	KX759621	**PP766459**	OQ709197	1.27E-03	NS	NS	NS	NS	NS	1	no	
sapo_rdp_align_nt	*Sapovirus*				**OQ818319**										
sapo_rdp_align_nt	*Sapovirus*	6726	6787	OP963622	**Unknown (PP766459)**	NC_033776	1.31E-03	NS	NS	NS	NS	NS	1	no	
sapo_rdp_align_nt	*Sapovirus*				**Unknown(OQ818319)**	KX759622									
sapo_rdp_align_nt	*Sapovirus*	925	1501	KX759619	Unknown (PP712001)	**PP766459**	1.88E-03	NS	3.84E-02	NS	NS	NS	2	no	
sapo_rdp_align_nt	*Sapovirus*			KX759623		**OQ818319**									
sapo_rdp_align_nt	*Sapovirus*	1111	1399	KX759623	ON872527	OQ709197	3.11E-03	NS	NS	3.16E-02	0.035006714	NS	3	no	
sapo_rdp_align_nt	*Sapovirus*			**OQ818319**											
sapo_rdp_align_nt	*Sapovirus*			**PP766459**											
sapo_rdp_align_nt	*Sapovirus*	1988	2076	OQ709197	Unknown (PP712004)	**PP766459**	1.40E-02	NS	NS	NS	NS	NS	1	no	
sapo_rdp_align_nt	*Sapovirus*					**OQ818319**									
sapo_rdp_align_nt	*Sapovirus*	4363	4565	KX759619	Unknown (PP712001)	**PP766459**	2.86E-02	NS	NS	NS	NS	NS	1	no	
sapo_rdp_align_nt	*Sapovirus*				**Unknown(OQ818319)**	PP712001									
sapo_rdp_align_nt	*Sapovirus*				**Unknown(PP766459)**										
tescho_rdp_align_nt_clade	*Teschovirus*	6968	7180	r_aegypticus_africa	**OQ818323**	**Unknown (OQ818324)**	1.04E-20	3.09E-07	4.54E-21	9.73E-11	6.32E-10	8.05E-09	6	yes	Supplementary Fig. 7E
tescho_rdp_align_nt_clade	*Teschovirus*				**OQ818324**	**Unknown(OQ818323)**									
tescho_rdp_align_nt_clade	*Teschovirus*	28	915	**OQ818323**	**OQ818324**	r_aegypticus_africa	4.70E-06	NS	5.47E-04	1.19E-08	1.76E-06	1.66E-04	5	yes	Supplementary Fig. 7D
tescho_rdp_align_nt_clade	*Teschovirus*	1128	1887	rousettus_china	Unknown (OR951324)	**OQ818318**	6.19E-03	NS	1.06E-02	3.34E-02	0.042472084	NS	4	yes	
tescho_rdp_align_nt_clade	*Teschovirus*	3299	6944	r_aegypticus_africa	**OQ818324**	**Unknown (OQ818323)**	2.12E-02	NS	NS	1.96E-04	NS	1.63E-04	3	no	
tescho_rdp_align_nt_clade	*Teschovirus*				**OQ818323**	**Unknown(OQ818324)**									
tescho_rdp_align_nt_clade	*Teschovirus*	6736	6849	**OQ818318**	rousettus_china	LC386158	NS	NS	4.14E-02	0.021570412	NS	NS	2	no	
tescho_rdp_align_nt	*Teschovirus*	5039	913	PP711934	**OQ818324**	**OQ818323**	2.33E-15	NS	6.56E-06	2.02E-04	5.00E-10	2.66E-14	5	yes	Fig. 5B
tescho_rdp_align_nt	*Teschovirus*	3231	5072	**OQ818323**	Unknown (OR951335)	PP711934	1.63E-07	NS	9.02E-08	2.11E-02	2.88E-10	NS	4	yes	
tescho_rdp_align_nt	*Teschovirus*	6692	6832	PP711948	**OQ818318**	LC386158	1.56E-04	NS	9.86E-03	NS	NS	9.78E-05	3	no	
tescho_rdp_align_nt	*Teschovirus*	539	749	OR951335	**Unknown (OQ818324)**	**OQ818323**	2.20E-03	NS	NS	3.81E-03	2.02E-02	NS	3	no	
tescho_rdp_align_nt	*Teschovirus*	6307	54	OR951335	Unknown (OR951333)	**OQ818323**	2.24E-03	NS	NS	4.51E-05	1.81E-02	NS	3	no	
tescho_rdp_align_nt	*Teschovirus*	3981	4236	OR951324	**OQ818323**	OR951334	4.02E-03	NS	NS	NS	NS	NS	1	no	
tescho_rdp_align_nt	*Teschovirus*			**OQ818318**											
tescho_rdp_align_nt	*Teschovirus*	3534	3708	**OQ818318**	PP711948	OR951334	NS	NS	1.87E-02	NS	NS	NS	1	no	
tescho_rdp_align_nt	*Teschovirus*	6284	6306	OR951335	**OQ818324**	OR951333	NS	NS	2.55E-02	NS	NS	NS	1	no	
tescho_rdp_align_nt	*Teschovirus*	74	354	OR951335	**OQ818324**	Unknown (OR951333)	NS	NS	2.24E-02	NS	2.17E-02	NS	2	no	
tescho_rdp_align_nt	*Teschovirus*	1965	2029	**OQ818323**	PP711934	**Unknown (OQ818318)**	2.56E-02	NS	NS	NS	NS	NS	1	no	
tescho_rdp_align_nt	*Teschovirus*	1482	1660	OR951333	OR951324	**OQ818324**	3.02E-02	NS	2.87E-02	0.044139616	NS	NS	3	no	
tescho_rdp_align_nt	*Teschovirus*			OR951334											
tescho_rdp_align_nt	*Teschovirus*	1237	1321	OR951335	**OQ818323**	**OQ818318**	3.22E-02	NS	NS	NS	NS	NS	1	no	
